# Progress in the study of bioactivity, chemical composition and pharmacological mechanism of action in *Wolfiporia cocos* (F.A. Wolf) Ryvarden & Gilb

**DOI:** 10.3389/fphar.2025.1521235

**Published:** 2025-03-03

**Authors:** Qi Xiong, Zhuoran Li, Defeng Yang, Xinze Liu, Wanxu Pu, Xitao Yue, Kaikai Jia, Xilin Wan, Yuanjun Zou

**Affiliations:** ^1^ School of Medical Information, Changchun University of Chinese Medicine, Changchun, China; ^2^ Department of Ophthalmology, The Second Hospital of Jilin University, Changchun, China; ^3^ Jilin Ginseng Academy, Changchun University of Chinese Medicine, Changchun, China; ^4^ Jilin Aodong Pharmaceutial Group Co., Ltd., Post-Doctoral Research Center, Yanji, China

**Keywords:** Wolfiporia cocos (F.A. Wolf) Ryvarden & Gilb, polysaccharide, triterpenoid metabolites, active metabolites, mechanism of action

## Abstract

The Latin name of *Wolfiporia cocos* is *Wolfiporia cocos* (F.A. Wolf) Ryvarden & Gilb, it a medicinal and edible mushroom belonging to the family Polyporaceae. Traditional Chinese medicine believes that it can strengthen the spleen, diuretic, tranquillise the mind and dispel dampness. So far, the chemical and active metabolites isolated and extracted from *Wolfiporia cocos* are mainly polysaccharides, triterpenoids, and sterols. Modern pharmacology has found that these chemical and active metabolites have a wide range of pharmacological effects, including antitumour, antioxidation, anti-inflammatory, immunomodulation, regulation of intestinal flora, regulation of glycolipid metabolism, and improvement of organ function. By applying *Poria cocos*, *Poria*, *Wolfiporia cocos, Wolfiporia cocos* (F.A. Wolf) Ryvarden & Gilb as search terms, we searched all the relevant studies on *Poria cocos* from Web of Science and PubMed databases and classified these categories of chemical and active metabolites according to the main research content of each literature and summarized its mechanism of action, updated its latest research results, and discussed the direction of further research in the future to provide a better reference for future clinical applications with better therapeutic effects and potential medicinal value.

## 1 Introduction

*Wolfiporia cocos* (F.A. Wolf) Ryvarden & Gilb. is the current accepted Latin name, and it formerly was known as *MacrohyWolfiporia cocos* (Schwein.) I. Johans. & Ryvarden., *Poria cocos* (syn. *Wolfiporia cocos*), *Poria cocos* F.A. Wolf, *Pachyma cocos* (Schwein.) Fr., and Sclerotium cocos Schwein (Li et al., 2022), which is known as “Fuling” in China and is now widely used in China, Japan and other parts of Asia. It is a healthcare edible mushroom belonging to the family Polyporaceae, which grows on the roots of pine trees in China (Nie et al., 2020). *Wolfiporia cocos* was first recorded in the famous Chinese medical book “*Shennong Bencao Jing*” and has been used for 2000 years (Li et al., 2019a). It is a kind of traditional Chinese medicine used for both food and medicine, which can strengthen the spleen, diuretic, tranquillize the mind and dispel dampness (Ng et al., 2024). Existing studies have shown that the active metabolites of *Wolfiporia cocos* are mainly triterpenoids, polysaccharides, sterols, and others, of which the active metabolites have biological functions such as antitumour (Li et al., 2024; Yue et al., 2023), regulation of intestinal flora (Lai et al., 2023), improvement of organ function (Jiang et al., 2022; Wu et al., 2023a), immunomodulation (Zhang W. et al., 2023), anti-inflammatory (Wu et al., 2023b), antioxidation (Fang et al., 2021), and regulation of glycolipid metabolism (Pan et al., 2023). By applying *Poria cocos*, *Poria*, *Wolfirporia cocos, Wolfiporia cocos* (F.A. Wolf) Ryvarden & Gilb as search terms, we searched all the relevant studies on *Wolfiporia cocos* from Web of Science and PubMed databases and classified these categories of chemical and active metabolites according to the main research content of each literature and summarized its mechanism of action, updated its latest research results, and discussed the direction of further research in the future to provide a better reference for future clinical applications with better therapeutic effects and potential medicinal value.

## 2 Active ingredients in *Wolfiporia cocos*

### 2.1 Polysaccharides

Polysaccharides refer to a class of high molecular weight metabolites, which are composed of more than 10 monosaccharides and are connected by glycosidic bonds. *Wolfiporia cocos* polysaccharides, as one of the main active ingredients of *Wolfiporia cocos*, account for about 84% of the active ingredients in *Wolfiporia cocos* sclerotia (Li et al., 2019b). *Wolfiporia cocos* polysaccharides can be divided into two categories based on their structure: glucans and heteropolysaccharides, with heteropolysac-charides mainly consisting of glucose, galactose, and mannose (Huang Q. et al., 2007). Chihara et al. (1970) extracted Pachyman from *Wolfiporia cocos*, which is mainly composed of β-(1→3)-D-glucan and also contains a small amount of β-(1→6) glycosidic side chains. Narui et al. (Narui et al., 1980) demonstrated through experiments that the structure of Pachyman extracted from *Wolfiporia cocos* mycelium cultured in the laboratory is almost identical to that extracted from *Wolfiporia cocos* grown in nature. The research results of Wang et al. (Wang et al., 2004) urther confirmed that the main component of *Wolfiporia cocos* polysaccharides is β-(1→3)-D-glucan. According to their solubility, *Wolfiporia cocos* polysaccharides are divided into water soluble polysaccharides (WPCP) whose backbone is composed of (1,6)-α-galactan and (1,3)-β-mannoglucan and alkaline soluble polysaccharides (APCP) whose backbone is composed of (1,3)-β-D-glucan (Zhao et al., 2023). Details are provided in Table 1.

**TABLE 1:** Polysaccharides from *Wolfiporia cocos*.

Components	Monosaccharide composition	Structural features	Pharmacological mechanism	References
H11	Glu	(1,3) -(1,6)-β-D-glucan	Antitumour	Kanayama et al. (1983)
PCS1	Fuc: Man: Gal: Glc = 9.2: 25.7: 47.9: 17.1	(1→3)-D-Glc-(1→6)-D-Glc; (1→6)-D-Gal, (1→4, 6)-D-Gal, (1→2, 6)-D-Man, (1→3,6)-D-Man	Not available	Wang et al. (2004)
PCS2	Fuc: Man: Gal: Glc = 1.5: 8.8: 6.5: 82.4	(1→3)-D-Glu, (1, terminal)-D-Glu, (1→6)-D-Glu, (1→2)-D-Gal, (1→3,6)-D-Man	Not available	Wang et al. (2004)
PCS3-I	Fuc: Xyl: Man: Gal: Glc = 9.0: 4.0: 39.3: 10.4: 37.2	Not available	Not available	Wang et al. (2004)
PCS3-II	Glc = 98.4	(1→3)-β-D-glucan with a linear	Not available	Wang et al. (2004)
PCS4-I	Fuc: Man: Glc = 1.2: 2.9: 93.1	(1→3)-β-D-glucan with some β-(1→6) and (1→2) linked branches	Not available	Wang et al. (2004)
PCS4-II	Glc = 97.2	(1→3)-β-D-glucan with some β-(1→6) and (1→2) linked branches	Not available	Wang et al. (2004)
wc-PCM0	Fuc: Ara: Xyl: Man: Gal: Glc = 4.1: 3: 2.5: 61.7	Not available	Antitumour	Jin et al. (2003a)
wc-PCM1	Fuc: Man: Gal: Glc = 10.5:24.5: 37.5: 30.6	Not available	Antitumour	Jin et al. (2003a)
wc-PCM2	Fuc: Man: Gal: Glc = 3.4: 12.5: 13.4: 70.7	Not available	Antitumour	Jin et al. (2003a)
wb-PCM0	Xyl: Glu: Ara:Man: Gal: Glc = 3.9: 71.1: 71.1: 6.1: 3.9: 11.4	(1,3)-α-D-glucan, β-D-mannose, β-D-galactose	Antitumour	Jin et al. (2003a)
wb-PCM1	Man: Glu: Gal = 7.7: 73.1: 19.2	Not available	Antitumour	Jin et al. (2003a)
wb-PCM3-I	Fuc: Ara: Man: Gal: Glc = 1.0: 2.2: 95.6: 20.5	(1→3)-α-D-glucan	Not available	Jin et al. (2003b)
wb-PCM3-II	Fuc: Ara: Xyl: Man: Gal: Glc = 2.6: 2.0: 1.2: 2.0: 91.4	(1→3)-β-D-glucan	Not available	Jin et al. (2003b)
wb-PCM4-I	Man: Glu = 5.8: 94.1	Not available	Not available	Jin et al. (2003b)
wb-PCM4-II	Glu: Gal = 76.1: 23.9	(1→3)-β-D-glucan	Not available	Jin et al. (2003b)
wc-PCM0	Fuc: Ara: Xyl: Man: Gal: Glc = 4.1: 3: 2.5: 61.7: 15	Not available	Not available	Jin et al. (2003b)
wc-PCM1	Fuc: Xyl: Man: Gal: Glc = 10.5: 24.5: 37.5: 30.6	Not available	Not available	Jin et al. (2003b)
wc-PCM2	Fuc: Xyl: Man: Gal: Glc = 3.4: 12.5: 13.4: 70.7	Not available	Not available	Jin et al. (2003b)
wc-PCM3-I	Xyl: Man: Glu = 6.4: 16.7: 76.9	Protein-bound (1→3)-β-D-glucan	Not available	Jin et al. (2003b)
wc-PCM3-II	Glu	Not available	Not available	Jin et al. (2003b)
wc-PCM4-I	Not available	Not available	Not available	Jin et al. (2003b)
wc-PCM4-II	Not available	Not available	Not available	Jin et al. (2003b)
ac-PCM0	Xyl: Man: Glc = 1.4: 1: 43	Not available	Antitumour	Jin et al. (2003a)
ac-PCM1	Fuc: Man: Gal: Glc = 4.5: 15.8: 23.9: 53.4	Not available	Antitumour	Jin et al. (2003a)
ac-PCM2	Fuc: Man: Gal: Glc = 0.8: 19.1: 29.7: 51.4	Not available	Antitumour	Jin et al. (2003a)
ab-PCM0	Man: Gal: Glc = 9.2: 11.1: 21.5	Not available	Antitumour	Jin et al. (2003a)
ab-PCM1	Fuc: Ara: Xyl: Man: Gal: Glc = 7.9: 4.0: 2.6: 10.5: 27.6: 47.3	Not available	Antitumour	Jin et al. (2003a)
ab-PCM2 - II	Man: Gal: Glc = 5.6: 13.1: 81.2	Not available	Antitumour	Jin et al. (2003a)
PCSC	Man: Gal: Ara = 92: 6.2: 1.3	Not available	Immunomodulation	Lee and Jeon (2003)
PCM3 - II	Glu	Not available	Antitumour	Zhang et al. (2006)
Pi-PCM0	Ara: Xyl: Man: Gal: Glc = 2.5: 1.5: 70.6: 18.5: 7	Not available	Antitumour	Huang et al. (2007b)
Pi-PCM1	Fuc: Ara: Xyl: Man: Gal: Glc = 10.9: 1.0: 2.8: 23.6: 36.5: 25.2	Not available	Antitumour	Huang et al. (2007b)
Pi-PCM2	Fuc: Man: Gal: Glc = 1.9: 29.6: 38.9: 29.7	Not available	Antitumour	Huang et al. (2007b)
Pi-PCM3-I	Glu	Not available	Not available	Huang et al. (2007b)
Pi-PCM3-II	Man: Gal: Glc = 10.9: 21.0: 68.1	Not available	Not available	Huang et al. (2007b)
Pi-PCM4-I	Glu	(1→3)-β-D-glucan	Not available	Huang et al. (2007b)
Pi-PCM4-II	Gal: Glc = 45.6: 54.4	(1→3)-β-D-glucan	Not available	Huang et al. (2007b)
PCP-I	Fuc: Man: Glc: Gal = 1: 1.81: 0.27: 7.27	Not available	Immunomodulation	Wu et al. (2016)
PCP-II	Fuc: Man: Glc: Gal = 1: 1.63: 0.16: 6.29	Not available	Immunomodulation	Wu et al. (2016)
PCWPW	Fuc: Man: Glc: Gal = 15.3: 36.8: 7.2: 40.4	Not available	Antidepressant/Immunomodulation	Zhang et al. (2023a)
PCWPS	Fuc: Man: Glc: Gal = 10.1: 30.07: 16.6: 41.47	Not available	Antidepressant/Immunomodulation	Zhang et al. (2023a)
CMP33	Glu	Not available	Antitumour	Liu et al. (2019)
CMP-1	Glu	(1→3)-β-D-glucan	Immunomodulation	Liu et al. (2021)
CMP-2	Man: Glc = 0.03:1	Not available	Immunomodulation	Liu et al. (2021)
PCP-1C	Fuc: Man: Gal: Glc = 14.6: 17.4: 43.5: 24.4	Not available	Anti-inflammatory	Cheng et al. (2021)
EPS - 0M	Glc: Man: Gal: Fuc: Rha = 17.3:46.3:19.9:8.7:5.0	Not available	Anti-inflammatory/Immunomodulation	Li et al. (2023)
EPS - 0.1M	Glc: Man: Gal: Fuc: Rha = 11.5:46.5:21.9:10.7:5.6	Not available	Anti-inflammatory/Immunomodulation	Li et al. (2023)
IPS - 0M	Glc: Man: Gal: Fuc: Rha = 79.7:8.9:5.5:1.7:3.1	Not available	Anti-inflammatory	Li et al. (2023)
IPS - 0.1M	Glc: Man: Gal: Fuc: Rha = 50.3:20.9:16.1:6.0:4.0	Not available	Anti-inflammatory/Immunomodulation	Li et al. (2023)

### 2.2 Triterpenoids

**FIGURE 1:**
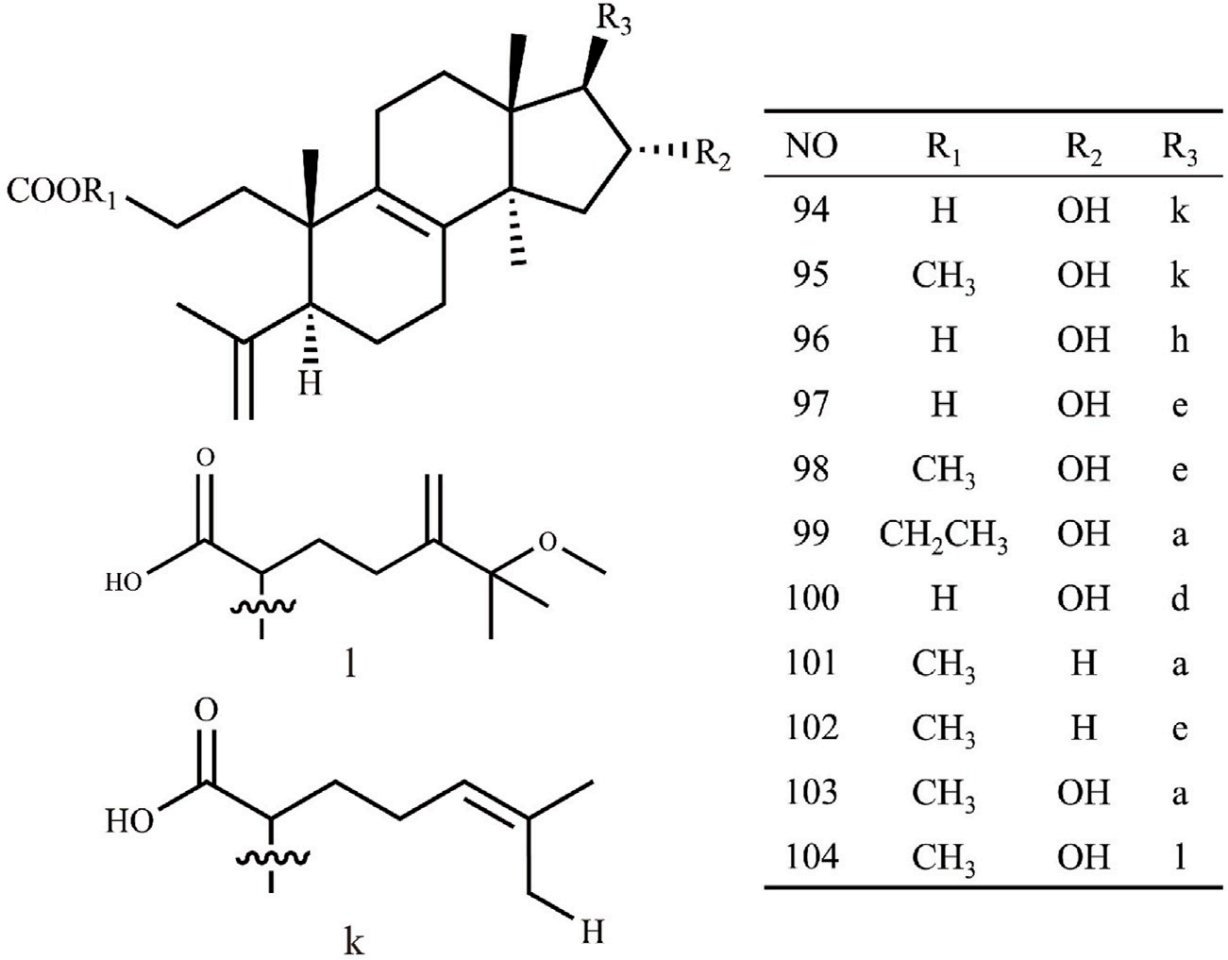
Structures of Lanosta-8-ene type triterpenes in *Wolfiporia cocos*.

**FIGURE 2:**
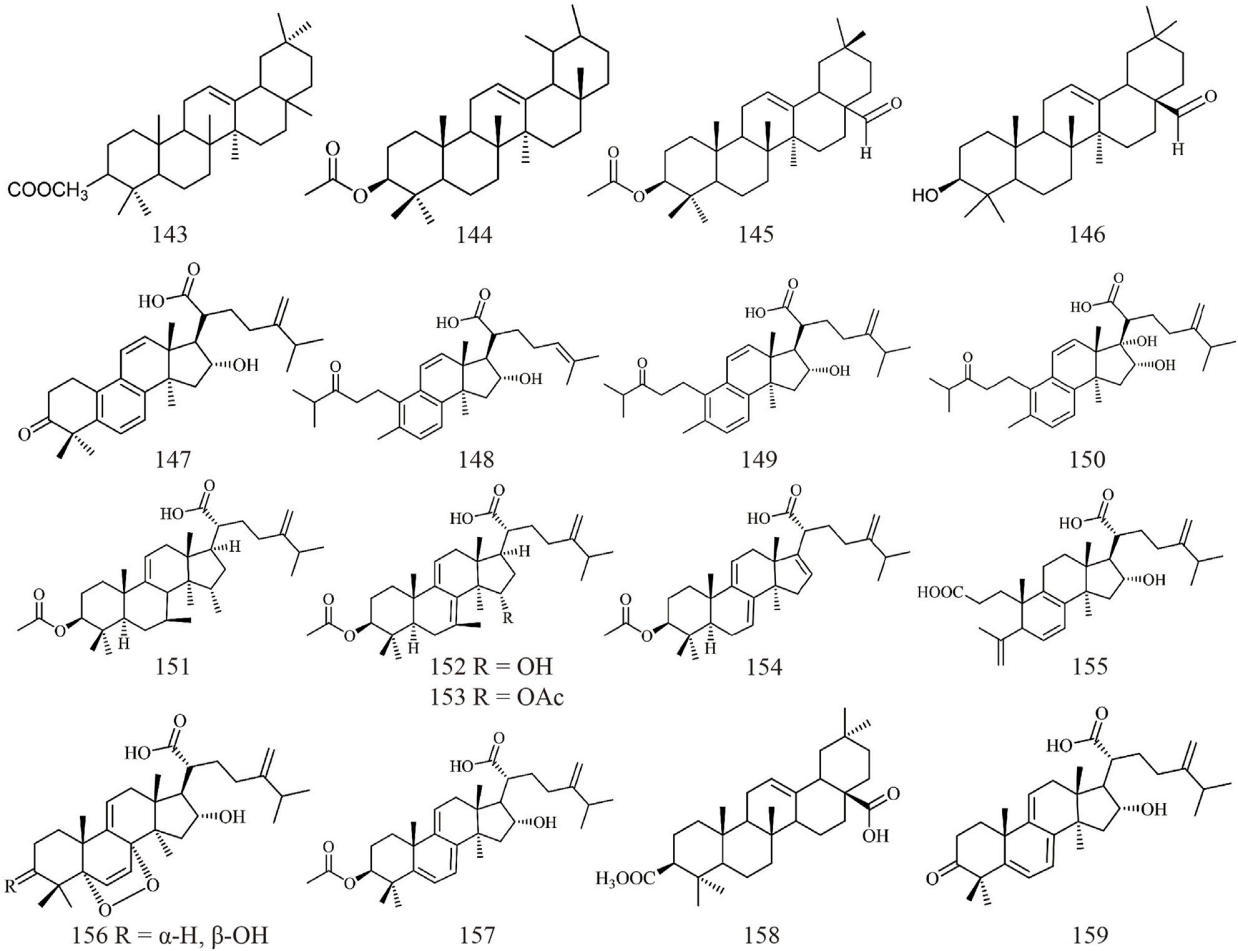
Structures of Lanosta-7,9(11)-diene type triterpenes in *Wolfiporia cocos*.

**FIGURE 3:**
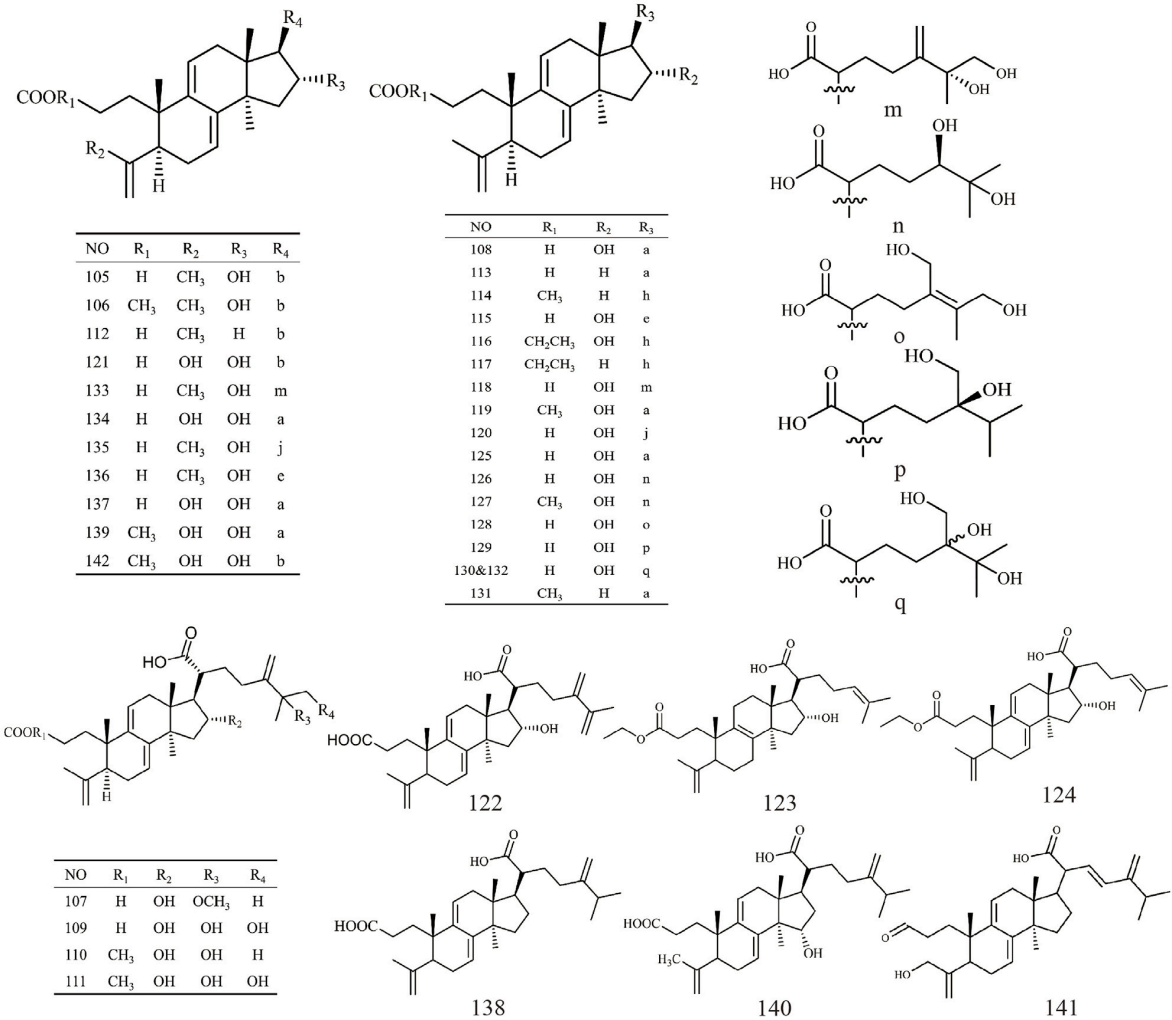
Structures of 3,4-seco-lanostan-8-ene type triterpenes in *Wolfiporia cocos*.

**FIGURE 4:**
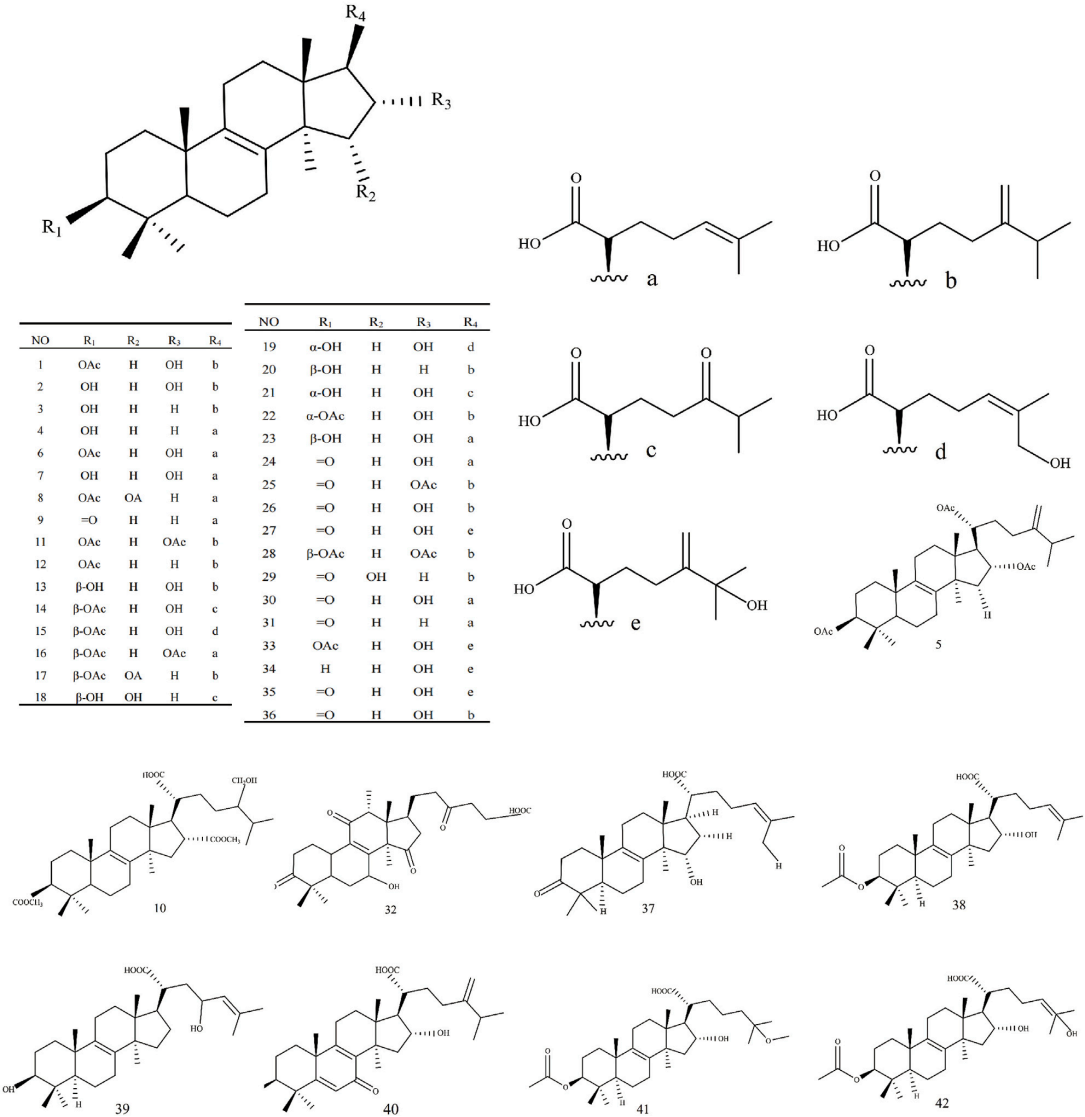
Structures of 3,4-seco-lanostan-7,9(11)-diene type triterpenes in *Wolfiporia cocos*.

**FIGURE 5:**
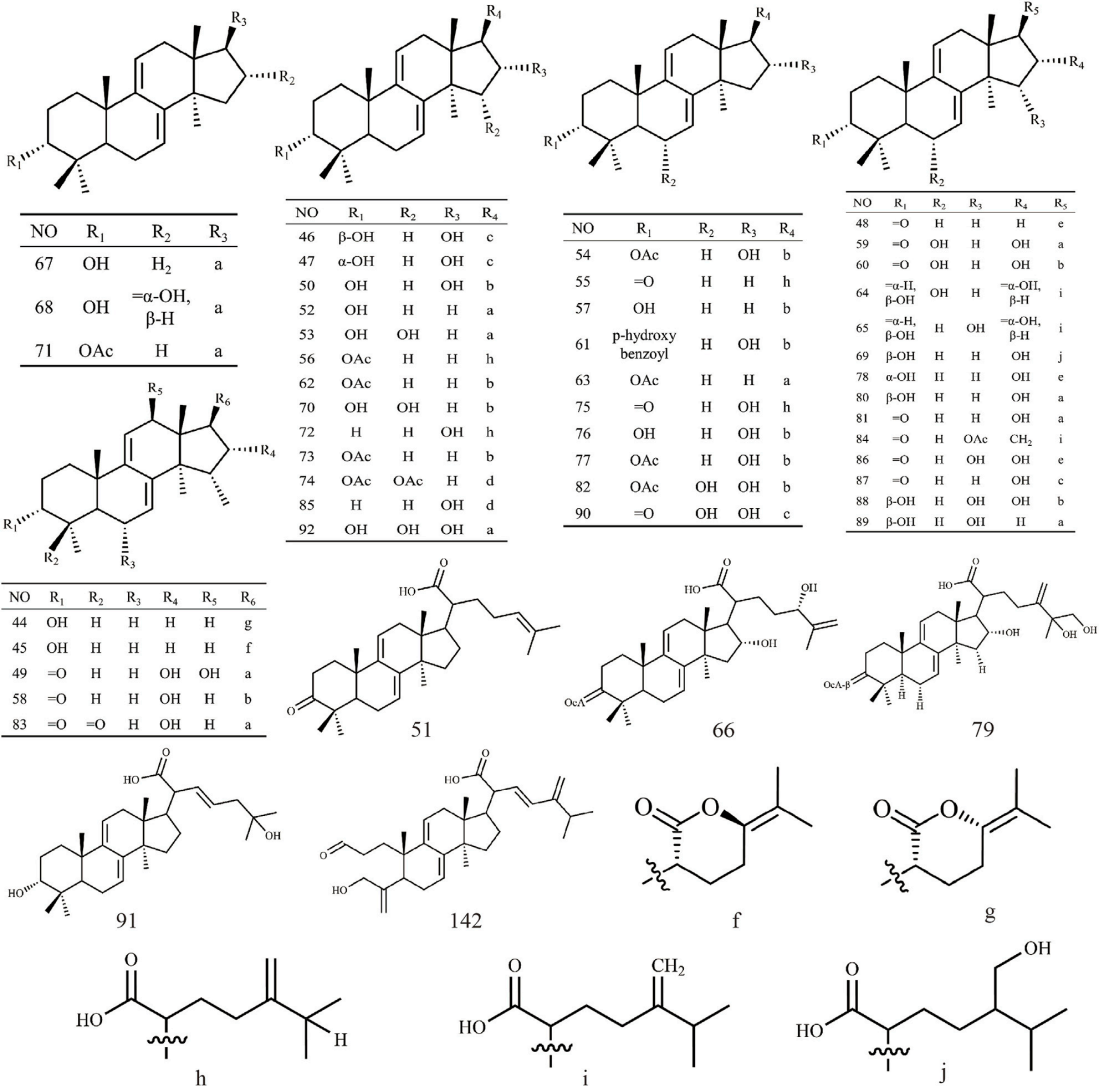
Structures of other type triterpenes in *Wolfiporia cocos*.

Triterpenoids, as one of the main active ingredients of *Wolfiporia cocos*, have a basic parent nucleus composed of 30 carbon atoms, and their structure can be regarded as a polymer of six isoprene units (Chen et al., 2018a). So far, more than 100 triterpenes with different skeletons have been discovered, among which pentacyclic triterpenes and tetracyclic triterpenes have the highest content (Andre et al., 2016). The triterpenoids in *Wolfiporia cocos* are mainly divided into two categories based on their number of rings: tetracyclic triterpenoids and pentacyclic triterpenoids, with tetracyclic triterpenoids dominating. We classified 159 triterpenoids obtained from the literature based on their different molecular backbone characteristics and grouped triterpenoids with similar molecular backbones. Details are provided in Table 2 and Figures 1–5.

**TABLE 2:** Triterpenoids from *Wolfiporia cocos*.

No	Chemical components	Formula	Molecular mass	Pharmacological properties	References
Lanosta-8-ene type triterpenes					
1	Pachymic acid	C_33_H_52_O_5_	527.37	Regulation of glycolipid metabolism, anti-inflammatory, antioxidation, inhibition of LDH and α-glucosidase activity	Li et al. (2017)
2	Tumulosic acid	C_31_H_50_O_4_	485.36	Anti-inflammatory	Fu et al. (2018)
3	Eburicoic acid	C_31_H_50_O_3_	470.72	Regulation of glycolipid metabolism, antioxidation, inhibition of LDH activity	Li et al. (2017)
4	Trametenolic acid	C_30_H_48_O_3_	456.7	Antioxidation, inhibition of LDH activity	Li et al. (2017)
5	Methyl pachymate	C_34_H_56_O_6_	560.8	Not available	Wang et al. (1993)
6	3-O-acetyl-16α-hydroxytrametenolic acid	C_32_H_50_O_5_	513.35	Inhibition α-glucosidase activity	Ma et al. (2023)
7	16α-hydroxytrametenolic acid	C_30_H_48_O_4_	471.34	Anti-inflammatory	Nukaya et al. (1996)
8	Versisponic acid E	C_35_H_54_O_5_	554.8	Regulation of glycolipid metabolism	Chen et al. (2019)
9	Oxotrametenolic acid	C_30_H_46_O_4_	470.68	Not available	Lee et al. (2017a)
10	O-acetylpachymic acid-25-ol	C_35_H_56_O_7_	588.81	Not available	Wang and Wan (1998)
11	O-acetylpachymic acid	C_35_H_54_O_6_	570.8	Not available	Wang et al. (1993)
12	Acetyl eburicoic acid	C_33_H_52_O_4_	512.76	Antitumour	León et al. (2004)
13	3β,16α-dihydroxy-7-oxo-24-methyllanosta-8,24(31)-dien-21-oic acid	C_31_H_48_O_5_	523.34	Not available	Lai et al. (2016)
14	3β-acetyloxy-16α-hydroxy-24-oxolanost-8-en-21-oic acid	C_32_H_50_O_6_	529.35	Not available	Zou (2019)
15	3β-acetyloxy-16α,26-dihydroxylanosta-8,24-dien-21-oic acid	C_32_H_50_O_6_	529.35	Not available	Zou (2019)
16	3β,16α-bis(acetyloxy)-29-hydroxylanosta-8,24-dien-21-oic acid	C_34_H_52_O_7_	571.36	Not available	Zou (2019)
17	3β,16α-bis(acetyloxy)-24-methylenelanost-8-en-21-oic acid	C_35_H_54_O_6_	569.38	Not available	Zou (2019)
18	3β,15α-dihydroxy-24-oxolanosta-8-en-21-oic acid	C_30_H_48_O_5_	487.34	Not available	Zou (2019)
19	3α,16α,25-trihydroxylanosta-8,24-dien-21-oic acid	C_30_H_48_O_5_	487.34	Not available	Zou (2019)
20	Hispindic acid B	C_31_H_50_O_4_	485.36	Not available	Zou (2019)
21	Daedaleanic acid B	C_30_H_48_O_5_	487.34	Not available	Zou (2019)
22	3-epi-pachymic acid	C_33_H_52_O_5_	527.37	Not available	Zou (2019)
23	16α-hydroxyeburiconic acid	C_31_H_48_O_4_	483.35	Not available	Zou (2019)
24	16α-hydroxy-3-oxolanosta-8,24-dien-21-oic acid	C_30_H_46_O_4_	469.33	Not available	Zou (2019)
25	16α-acetyloxyeburiconic acid	C_33_H_50_O_5_	525.35	Not available	Zou (2019)
26	16α,29-dihydroxyeburiconic acid	C_31_H_48_O_5_	499.34	Not available	Zou (2019)
27	16α,25-dihydroxydehydroeburiconic acid	C_31_H_48_O_5_	499.34	Not available	Zou (2019)
28	16-O-acetylpachymic acid	C_35_H_54_O_6_	569.38	Not available	Zou (2019)
29	15α-hydroxyeburiconic acid	C_31_H_48_O_4_	483.34	Not available	Zou (2019)
30	Pinicolic acid E	C_30_H_46_O_4_	470.68	Regulation of glycolipid metabolism	Chen et al. (2019)
31	Pinicolic acid A	C_30_H_46_O_3_	454.68	Stimulating glucose uptake and improving insulin sensitivity, antibacterial	Chen et al. (2019)
32	Ganoderic acid	C_30_H_44_O_7_	516.66	Not available	Wang and Wan (1998)
33	25-hydroxypachymic acid	C_33_H_52_O_6_	544.76	Not available	Zheng and Yang (2008)
34	25-hydroxy-3-epitumulosic acid	C_31_H_49_O_5_	501.72	Inhibition of TPA-induced EBV-EA, cytotoxicity to HL60	Akihisa et al. (2009)
35	16α,25-dihydroxyeburicoic acid	C_31_H_47_O_5_	499.7	Inhibition of TPA-induced EBV-EA, cytotoxicity to CRL1579	Akihisa et al. (2009)
36	16α-hydroxyeburicoic acid	C_20_H_28_O_4_	332.43	Not available	Akihisa et al. (2009)
37	15α-hydroxy-3-oxolanosta-8,24-dien-21-oic acid	C_30_H_46_O_4_	469.33	Not available	Zou et al. (2019)
38	3β-ethanoyl-16α,23-dihydroxy-lanosta-8(9),24(25)-diene-21-oic acid	C_32_H_50_O_6_	553.35	Not available	Wang (2019)
39	3β,23-dihydroxy-lanosta-8(9),24(25)-diene-21-oic acid	C_30_H_49_O_4_	473.36	Not available	Wang (2019)
40	3α,16α-dihydroxy-7-oxo-lanosta-5(6),8(9),24(31)-trien-21-oic acid	C_31_H_46_O_5_	521.32	Not available	Wang (2019)
41	Ceanphytamic acid B	C_33_H_53_O_6_	545.77	Antitumour	Chen et al. (2018a)
42	Ceanphytamic acid A	C_32_H_49_O_6_	529.73	Antitumour	Chen et al. (2018a)
43	3-O-formyleburicoic acid	Not available	Not available	Not available	Hui et al. (2016)
Lanosta-7,9(11)-diene type triterpenes					
44	Porilactone B	C_30_H_45_O_3_	453.34	Stimulating glucose uptake and improving insulin sensitivity	Chen et al. (2019)
45	Porilactone A	C_30_H_45_O_3_	453.33	Stimulating glucose uptake and improving insulin sensitivity	Chen et al. (2019)
46	Poriacosones B	C_30_H_46_O_5_	485.32	Not available	Zheng and Yang (2008)
47	Poriacosones A	C_30_H_46_O_5_	485.32	Not available	Zheng and Yang (2008)
48	Polyporenic acid C	C_31_H_46_O_4_	481.33	Regulation of glycolipid metabolism, Cytotoxic to K562, anti-inflammatory, Antitumour	Zheng and Yang (2008)
49	Pinicolic acid F	C_30_H_47_O_6_	503.34	Stimulating glucose uptake and improving insulin sensitivity	Chen et al. (2019)
50	Dehydrotumulosic acid	C_31_H_48_O_4_	483.35	Anti-inflammatory, inhibition α-glucosidase activity	Ma et al. (2023)
51	Dehydrotrametenonic acid	C_30_H_44_O_3_	452.67	Not available	Akihisa et al. (2004)
52	Dehydrotrametenolic acid	C_30_H_46_O_3_	453.34	Anti-inflammatory, antioxidation, inhibition of LDH activity	Akihisa et al. (2004)
53	Dehydrosulphurenic acid	C_33_H_50_O_6_	542.74	Anti-inflammatory	Dong et al. (2015)
54	Dehydropachymic acid	C_33_H_50_O_5_	526.75	Stimulating glucose uptake and improving insulin sensitivity, anti-inflammatory, antioxidation, inhibition of LDH activity, Antitumour	Li et al. (2017)
55	Dehydroeburiconic acid	C_33_H_50_O_5_	526.75	Antitumour	Tai et al. (1995)
56	Dehydroeburicoic acid monoacetate	C_33_H_50_O_4_	510.75	Antitumour	Lai et al. (2016)
57	Dehydroeburicoic acid	C_33_H_50_O_3_	494.75	Anti-inflammatory, Antitumour	Fu et al. (2018)
58	6α-hydroxypolyporenic acid C	C_31_H_46_O_5_	498.69	Not available	Wang (2019)
59	6,16α-dihydroxydehydrotrametenonic acid	C_30_H_44_O_5_	483.31	Not available	Zou (2019)
60	6,16α-dihydroxydehydroeburiconic acid	C_31_H_46_O_5_	497.32	Not available	Zou (2019)
61	3β-p-hydroxybenzoyldehydrotumulosic acid	C_38_H_52_O_6_	603.36	Anti-inflammatory	Yasukawa et al. (1998)
62	3β-hydroxy-16α-acetoxy-lanosta-7,9(11),24-trien-21-oic acid	C_32_H_48_O_5_	511.34	Not available	Zou et al. (2019)
63	3β-acetoxylanosta-7,9(11),24-trien-21-oic acid	C_32_H_48_O_4_	496.72	Cytotoxic to K562	Lai et al. (2016)
64	3β,16α,29-trihydroxy-24-methyllanosta-7,9(11),24(31)-trien-21-oic acid	C_32_H_48_O_5_	523.33	Not available	Lai et al. (2016)
65	3β,16α,30-trihydroxy-24-methyllanosta-7,9(11),24(31)-trien-21-oic acid	C_32_H_48_O_5_	523.33	Not available	Lai et al. (2016)
66	3β-acetoxy-16α,24β-dihydroxylanosta-7,9(11),25-trien-21-oic acid	C_32_H_48_O_6_	551.33	Not available	Lai et al. (2016)
67	Lanosta-7,9(11),24-trien-21-oic acid	C_31_H_48_O_2_	452.71	Antitumour	Lai et al. (2016)
68	3β,16α-dihydroxylanosta-7,9(11),24-trien-21-oic acid	C_30_H_46_O_4_	470.68	Anti-inflammatory	Akihisa et al. (2004)
69	3β,16α-dihydroxy-24-hydroxymethyllanosta-7,9(11)-dien-21-oic acid	C_31_H_50_O_5_	501.35	Not available	Zou (2019)
70	3β,15α-dihydroxylanosta-7,9(11),24-triene-21-oic acid	C_31_H_48_O_4_	484.71	Not available	Dong et al. (2015)
71	3-O-acetyl-16α-hydroxy-dehydrotrametenolic acid	C_32_H_48_O_5_	511.34	Not available	Tai et al. (1995)
72	3-epidehydrotumulosic acid	C_31_H_48_O_4_	484.71	Not available	Tai et al. (1995)
73	3-epidehydropachymic acid	C_31_H_48_O_4_	484.71	Inhibition α-glucosidase activity	Ma et al. (2023)
74	3,15-O-diacetyl-dehydrotrametenolic Acid	C_34_H_50_O_6_	577.35	Not available	Chen et al. (2019)
75	29-hydroxypolyporenic acid C	C_31_H_46_O_5_	498.69	Not available	Zheng and Yang (2008)
76	29-hydroxydehydrotumulosic acid	C_31_H_48_O_5_	499.34	Anti-inflammatory	Cai and Cai (2011)
77	29-hydroxydehydropachymic acid	C_33_H_50_O_6_	541.35	Anti-inflammatory	Cai and Cai (2011)
78	25-hydroxy-3-epi-dehydrotumulosic acid	C_32_H_50_O_5_	514.73	Not available	Tai et al. (1995)
79	25,26-dihydroxydehydropachymic acid	C_33_H_50_O_7_	557.34	Not available	Zou (2019)
80	16α-hydroxydehydrotrametenolic acid	C_30_H_46_O_4_	469.33	Not available	Zou (2019)
81	16α-hydroxydehydrotrametenonic acid	C_30_H_44_O_4_	467.31	Not available	Zou (2019)
82	16α-hydroxydehydropachymic acid	C_33_H_50_O_6_	542.74	Anti-inflammatory	Nukaya et al. (1996)
83	16α-hydroxy-3-oxolanosta-7,9(11),24-trien-21-oic acid	C_30_H_44_O_4_	468.67	Not available	Chen et al. (2019)
84	16α-acetyloxy- 24-methylene-3-oxolanosta-7,9(11)-dien-21-oic acid	C_33_H_48_O_5_	523.34	Not available	Zou et al. (2019)
85	16α,27-dihydroxydehydrotrametenoic acid	C_30_H_46_O_5_	485.33	Inhibition of TPA-induced EBV-EA	Akihisa et al. (2009)
86	16α,25-dihydroxydehydroeburiconic acid	C_31_H_46_O_5_	497.33	Not available	Zou (2019)
87	16-hydroxy-3,24-dioxolanosta-7,9(11)-dien-21-oic acid	C_30_H_44_O_5_	483.31	Not available	Zou (2019)
88	15α-hydroxydehydrotumulosic acid	C_31_H_48_O_5_	499.34	Inhibition of TPA-induced EBV-EA	Akihisa et al. (2007)
89	15α-hydroxydehydrotrametenolic acid	C_30_H_46_O_4_	469.33	Not available	Zou (2019)
90	Poricoic acid ZI	C_30_H_43_O_6_	499.31	Not available	Wang (2019)
91	Poricoic acid ZE	C_30_H_46_O_4_	493.33	Anti-renal fibrosis	Wang (2019)
92	Poricoic acid ZL	C_30_H_47_O_5_	487.34	Not available	Wang (2019)
93	3-O-formyl-dehydrotrametenolic acid	Not available	Not available	Not available	Hui et al. (2016)
3,4-seco-lanostan-8-ene type triterpenes					
94	Poricoic acid G	C_30_H_46_O_5_	485.33	Cytotoxicity to HL60	Mizushina et al. (2004)
95	Poricoic acid GM	C_31_H_47_O_5_	499.7	Inhibition of TPA-induced EBV-EA	Akihisa et al. (2009)
96	Poricoic acid H	C_31_H_48_O_5_	499.34	Cytotoxicity to HL60	Mizushina et al. (2004)
97	Poricoic acid HM	C_32_H_49_O_5_	513.73	Inhibition of TPA-induced EBV-EA	Akihisa et al. (2009)
98	25-hydroxyporicoic acid H	C_30_H_48_O_6_	504.7	Not available	Akihisa et al. (2007)
99	Poricoic acid GE	C_30_H_46_O_5_	486.68	Not available	Dong et al. (2015)
100	Poricoic acid ZA	C_30_H_46_O_6_	502.68	Anti-renal fibrosis	Wang et al. (2017)
101	Poricoic acid ZJ	C_31_H_48_O_5_	523.34	Not available	Wang (2019)
102	Poricoic acid ZK	C_31_H_47_O_4_	483.34	Not available	Wang (2019)
103	Poricoic acid ZR	C_31_H_48_O_6_	539.33	Not available	Wang (2019)
104	25-methoxy-29-hydroxyporicoic acid HM	C_33_H_52_O_7_	559.36	Not available	Zou (2019)
3,4-seco-lanostan-7,9(11)-diene type triterpenes					
105	Poricoic acid A	C_31_H_46_O_5_	497.32	Antitumour, inhibition α-glucosidase and activity	Ma et al. (2023)
106	Poricoic acid AM	C_32_H_48_O_5_	512.72	Inhibition of TPA-induced EBV-EA	Tai et al. (1993)
107	25-methoxyporicoic acid A	C_32_H_48_O_6_	527.33	Inhibition of TPA-induced EBV-EA, Antitumour	Akihisa et al. (2009)
108	Poricoic acid B	C_30_H_44_O_5_	483.31	Antitumour, inhibition α-glucosidase activity	Ma et al. (2023)
109	25-hydroxyporicoic acid C	C_31_H_45_O_5_	497.68	Inhibition of TPA-induced EBV-EA, cytotoxicity to HL60	Akihisa et al. (2009)
110	Poricoic acid DM	C_32_H_48_O_6_	527.33	Inhibition of TPA-induced EBV-EA	Tai et al. (1993)
111	26-hydroxyporicoic acid DM	C_32_H_48_O_7_	544.72	Inhibition of TPA-induced EBV-EA	Akihisa et al. (2009)
112	Poricoic acid C	C_31_H_46_O_4_	481.33	Inhibition α-glucosidase activity	Ma et al. (2023)
113	16-deoxyporicoic acid B	C_30_H_44_O_4_	467.32	Antitumour	Akihisa et al. (2007)
114	Poricoic acid CM	C_32_H_48_O_4_	496.72	Inhibition of TPA-induced EBV-EA	Akihisa et al. (2007)
115	Poricoic acid D	C_31_H_46_O_6_	513.32	Stimulating glucose uptake and improving insulin sensitivity	Tai et al. (1993)
116	Poricoic acid AE	C_33_H_50_O_5_	526.75	Not available	Yang et al. (2009)
117	Poricoic acid CE	C_33_H_50_O_4_	510.75	Not available	Yang et al. (2009)
118	Poricoic acid L	C_31_H_46_O_7_	553.31	Stimulating glucose uptake and improving insulin sensitivity	Chen et al. (2019)
119	Poricoic acid BM	C_31_H_46_O_5_	498.69	Not available	Tai et al. (1995)
120	Poricoic acid E	C_30_H_44_O_6_	500.67	Not available	Tai et al. (1995)
121	Poricoic acid F	C_30_H_47_O_6_	503.34	Not available	Chen et al. (2019)
122	16α-hydroxy-3,4-secolanosta-4(28),7,11(9),24(31),25(27)-pentaene- 3,21-dioic acid	C_31_H_44_O_5_	495.31	Not available	Dong et al. (2017)
123	16α-hydroxy-3,4-seco-lanosta-4(28),8,24-triene-3,21-dioic acid-3-ethyl ester	C_32_H_50_O_5_	513.36	Not available	Dong et al. (2017)
124	16α-hydroxy-3,4-seco-lanosta-4(28),7(9),11,24-tetraene-3,21-dioic acid-3-ethyl ester	C_32_H_48_O_5_	511.34	Not available	Dong et al. (2017)
125	Poricoic acid I	C_31_H_47_O_6_	515.33	Regulation of glycolipid metabolism	Chen et al. (2019)
126	Poricoic acid J	C_31_H_47_O_7_	531.33	Stimulating glucose uptake and improving insulin sensitivity	Chen et al. (2019)
127	Poricoic acid JM	C_32_H_49_O_7_	545.34	Regulation of glycolipid metabolism	Chen et al. (2019)
128	Poricoic acid K	C_31_H_47_O_7_	533.34	Regulation of glycolipid metabolism	Chen et al. (2019)
129	Poricoic acid M	C_30_H_46_O_7_	541.31	Regulation of glycolipid metabolism	Chen et al. (2019)
130	Poricoic acid N	C_31_H_48_O_8_	571.32	Stimulating glucose uptake and improving insulin sensitivity	Chen et al. (2019)
131	16-deoxyporicoic acid BM	C_31_H_47_O_4_	483.35	Not available	Chen et al. (2019)
132	Poricoic acid O	C_31_H_48_O_8_	571.32	Stimulating glucose uptake and improving insulin sensitivity	Chen et al. (2019)
133	Poricoic acid ZB	C_31_H_46_O_7_	553.31	Not available	Wang (2019)
134	Poricoic acid ZC	C_30_H_44_O_6_	523.3	Anti-renal fibrosis	Wang (2019)
135	Poricoic acid ZD	C_31_H_47_O_7_	531.33	Anti-renal fibrosis	Wang (2019)
136	Poricoic acid ZG	C_30_H_46_O_6_	525.31	Antifibrotic	Chen et al. (2019)
137	Poricoic acid ZM	C_30_H_46_O_6_	525.31	Not available	Wang (2019)
138	Poricoic acid ZO	C_31_H_44_O_4_	503.31	Not available	Wang (2019)
139	Poricoic acid ZP	C_31_H_45_O_6_	513.32	Not available	Wang (2019)
140	Poricoic acid ZN	C_31_H_46_O_5_	521.32	Not available	Wang (2019)
141	Poricoic acid ZV	C_30_H_46_O_4_	493.33	Not available	Wang (2019)
142	Poricoic acid ZQ	C_32_H_48_O_6_	551.33	Not available	Wang (2019)
Other type triterpenes					
143	Β-amyrin acetate	C_32_H_52_O_2_	468.75	Not available	Wang and Wan (1998)
144	Α-amyrin acetate	C_32_H_52_O_2_	468.75	Not available	Yang et al. (2019)
145	Oleanolic acid 3-O-acetate	C_32_H_50_O_4_	498.73	Not available	Yang et al. (2019)
146	Oleanolic acid	C_30_H_48_O_3_	456.7	Not available	Dianpeng et al. (1998)
147	Daedaleanic acid F	C_31_H_43_O_4_	479.31	Regulation of glycolipid metabolism	Chen et al. (2019)
148	Daedaleanic acid E	C_30_H_42_O_4_	489.3	Stimulating glucose uptake and improving insulin sensitivity	Chen et al. (2019)
149	Daedaleanic acid D	C_31_H_45_O_4_	481.33	Stimulating glucose uptake and improving insulin sensitivity	Chen et al. (2019)
150	Daedaleanic acid A	C_31_H_46_O_4_	482.69	Stimulating glucose uptake and improving insulin sensitivity	Chen et al. (2019)
151	Coriacoic acid D	C_35_H_52_O_7_	584.78	Not available	Lee et al. (2017b)
152	Coriacoic acid C	C_35_H_50_O_5_	550.77	Not available	Lee et al. (2017b)
153	Coriacoic acid B	C_35_H_52_O_6_	568.78	Not available	Lee et al. (2017b)
154	Coriacoic acid A	C_33_H_48_O_4_	508.73	Not available	Lee et al. (2017b)
155	6,7-dehydroporicoic acid H	C_31_H_45_O_5_	497.68	Inhibition of TPA-induced EBV-EA	Akihisa et al. (2009)
156	5α,8α-peroxydehydrotumulosic acid	C_31_H_46_O_6_	513.32	Not available	Akihisa et al. (2007)
157	3β-acetyloxy-16α-hydroxy-24-methy-lenelanosta-5,7(9),11-tetraene-21-oic acid	C_33_H_48_O_5_	523.34	Not available	Dong et al. (2017)
158	3-acetoxy oleanolic acid	C_32_H_52_O_4_	500.75	Not available	Yang et al. (2014)
159	16α-hydroxy-3-oxo-24-methyllanosta-5,7,9(11),24(31)-tetraen-21-oic acid	C_31_H_44_O_4_	503.31	Not available	Lai et al. (2016)

### 2.3 Sterols

Sterol metabolites are a class of steroids, all of which have cyclopentane dihydrophenanthrene as their basic structure and are steroids containing hydroxyl groups (Yalcinkaya et al., 2024). Sterol metabolites mainly contain ergosterol and pregnancy sterols (Chen et al., 2018b). The representative metabolites of ergosterols mainly include ergosta-7.22-dien-3β-ol,ce-revisterol,ergosta-7-en-3β-ol (Jinming et al., 2001), β-sitosterol (Tong et al., 2010) and stigmas-terol (Ni et al., 2019). Representative metabolites of pregnancy sterols include pregn-7-ene-2β,3a,15a,20-tetrol and pregna-7-en-3a,11a,15a,20-quad-roil (Chen et al., 2018b). Details are provided in Table 3.

**TABLE 3:** Sterols from *Wolfiporia cocos*.

Chemical components	Formula	Molecular mass	References
Ergosterol	C_28_H_44_O	396.65	Yaoita et al. (2002)
(22E) -ergosta-5, 7, 9(11),22-tetraen-3β-ol	C_28_H_44_O	396.65	Yaoita et al. (2002)
Ergosta-5, 7-dien-3β-ol	C_28_H_44_O	396.65	Yaoita et al. (2002)
(22E) -ergosta-8(14),22-dien-3β-ol	C_28_H_46_O	398.66	Yaoita et al. (2002)
(22E) -ergosta-6, 8(14),22-trien-3β-ol	C_28_H_44_O	396.65	Yaoita et al. (2002)
(22E) -ergosta-7, 22-dien-3β-ol	C_28_H_46_O	398.66	Yaoita et al. (2002)
Ergost-7-en-3β-ol	C_28_H_48_O	400.68	Yaoita et al. (2002)
Ergosterol peroxide	C_28_H_44_O_3_	428.65	Li et al. (2004)
Pregn-7-ene-2β, 3α, 15α, 20-tetrol	C_21_H_34_O_4_	350.49	Chen et al. (2018b)
3β,5α-dihydroxy-ergosta-7,22-dien-6-one	C_28_H_46_O_3_	430.66	Yang et al. (2014)
3β,5α,9α-trihydroxy-ergosta-7,22-diene-6one	C_28_H_46_O_4_	446.66	Yang et al. (2014)
Ergosta-7,22-diene-3-one	C_28_H_44_O	396.65	Yang et al. (2014)
6,9-epoxy-ergosta-7,22-diene-3-ol	C_28_H_46_O_2_	414.66	Yang et al. (2014)
Ergosta-4,22-diene-3one	C_28_H_46_O	398.66	Yang et al. (2014)
Ergosta-5,6-epoxy-7,22-dien-3-ol	C_28_H_46_O_2_	414.66	Yang et al. (2014)
Preg-7-ene-2β,3α,15α,20-tetrol	C_21_H_31_O_4_	347.47	Tong et al. (2010)
Β-sitosterol	C_31_H_52_O_2_	456.74	Tong et al. (2010)
9,11 - dehydroergosterol peroxide	C_28_H_44_O_3_	428.65	Lee et al. (2018)

### 2.4 Other ingredients

In addition to polysaccharides, triterpenoids, and sterols, there are also some other types of chemical metabolites in *Wolfiporia cocos*. Such as tricyclic diterpenes (Shen et al., 2012) and sohiracillinone (Chen et al., 2018a). Organic acids and their esters include protocatechuic acid, palmitic acid, ethyl palmitate, methyl palmitate, trimethyl citrate, dimethyl(R)-malate, di-(2-ethylhexyl) phthalate, dibutyl phthalate, octadecanoic acid, octacosyl acid and pentacosanoic acid (Yang et al., 2019). In addition, 51 proteins were isolated and identified from the fermentation broth of *Wolfiporia cocos*. Some studies have found that volatile oil metabolites from *Wolfiporia cocos* (Jie et al., 2014) contain abundant trace elements required by the human body, such as iron, zinc, manganese, potassium, sodium, selenium, calcium and phosphorus. Among them, iron has the highest content, followed by zinc and manganese (Xi and Zhang, 2022).

## 3 Pharmacological mechanism of active ingredients in *Wolfiporia cocos*

### 3.1 Antitumour activity

A large number of studies have found that the anticancer effect of the active ingredients in *Wolfiporia cocos* on lung cancer (Jiang and Duanmu, 2021), breast cancer (Jeong et al., 2015), gastric cancer (Lu et al., 2018), liver cancer (Huang et al., 2006), pancreatic cancer (Cheng et al., 2013), and kidney cancer (Li et al., 2024) may inhibit tumor cell proliferation and metastasis and induce tumor cell apoptosis by regulating some signal pathways and the expression level of tumor-related cytokines.

Recent pharmacological studies have uncovered the antitumor mechanisms associated with bioactive components derived from *Wolfiporia cocos*. Pachymic acid (PA) has been shown to disrupt tumor cell architecture and induce apoptosis in renal tumor cells *via* upregulation of tumor protein p53-inducible nuclear protein 2 (TP53INP2) and tumor necrosis factor receptor-associated factor 6 (TRAF6), alongside activation of pro-apoptotic pathways involving caspase-8, caspase-3, and PARP (Li et al., 2024). Chen et al. (2015) demonstrated that PA inhibits migration and invasion of gallbladder cancer cells in a dose-dependent manner by downregulating tumor-associated proteins including PCNA, ICAM-1, RhoA, p-Akt, and p-ERK1/2, mediated through inhibition of the AKT and ERK pathways. Ling et al. (2011) showed that PA suppresses invasion and metastasis of MDA-MB-231 and MCF-7 breast cancer cells by inhibiting the NF-κB signaling pathway and MMP-9 activity. Wang et al. (2022) demonstrated that PA inhibits gastric cancer (GC) cell viability and proliferation in a concentration-dependent manner. This reduction in GC cell adhesion effectively hampers metastasis and invasion. PA also significantly alters the expression of epithelial-mesenchymal transition (EMT)-related proteins, including E-cadherin, N-cadherin, and Vimentin, while concurrently decreasing the levels of metastasis-related proteins, including matrix metalloproteinases MMP-2 and MMP-9, along with tissue inhibitors of metalloproteinase 1.

Chen et al. (2022) demonstrated that poricoic acid A (PAA) exhibits significant therapeutic effects on T-cell acute lymphoblastic leukemia (T-ALL). Both *in vitro* and *in vivo* models showed that PAA markedly reduced T-ALL cell viability, induced G2 phase cell cycle arrest, and triggered apoptosis by exacerbating mitochondrial dysfunction and generating excessive reactive oxygen species (ROS). Additionally, PAA was found to induce autophagy and ferroptosis in T-ALL cells by regulating the AMPK/mTOR and LC3 signaling pathways, thus amplifying its therapeutic effects. Ma et al. (2021a) reported that PAA triggers apoptosis in SKOV3 ovarian cancer cells through mitochondrial and death receptor pathways in a concentration-dependent manner. Its antitumor mechanisms involve inhibition of the mTOR/p70S6K signaling pathway, an increase in LC3-I and LC3-II protein levels, activation of caspase-3, caspase-8, and caspase-9, and modulation of pro-apoptotic and anti-apoptotic protein expression.

Jiang et al. (2022) discovered that *Wolfiporia cocos* polysaccharides can dose-dependently inhibit the proliferation of lung cancer cells and suppress the migration and invasion of A549 cells by downregulating MMP-2 and MMP-9 through inhibition of the NF-κB signaling pathway. Moreover, neutral polysaccharide metabolites (Chen and Chang, 2004) and triterpenoids (Ukiya et al., 2002) isolated from *Wolfiporia cocos* have been reported to inhibit the proliferation and differentiation of HL-60 human leukemia cells. Lin et al. (Lin et al., 2020) discovered that the fucose-containing mannoglucan polysaccharide (FMGP) extracted from *Wolfiporia cocos* significantly inhibits the metastasis of CL1-5 lung cancer cells. FMGP achieves this by inhibiting the TGFβ RI/FAK/AKT signaling pathway and reducing the expression of the metastasis-associated protein Slug. Table 4 summarizes the antitumor bioactivities of *Wolfiporia cocos* extraction.

**TABLE 4:** Antitumor activities in *Wolfiporia cocos* extraction.

Model used	Extracts metabolites	Cancer type	Cell line	Human/Mice cell	Activities	Dose range tested	Duration	Minimal active concentration	Control	Sample sources	References
*In vivo*/*In vitro*	Poricoic acid A	Leukemia	T-ALL	Human	↑ROS, ↑MDA, ↓GSH.	*In vivo*: low dose of PAA (5 mg/kg) and high dose of PAA (10 mg/kg), *In vitro*:1.25 μM–50 μM	*In vivo*-4 weeks *In vitro*-24 h	IC50: JURKAT: 4.31 μM MOLT-3: 10.73 μM ALL-SIL: 8.89 μM RPMI-8402: 11.21 μM	Negative	*Wolfiporia cocos* surface layer	Chen et al. (2022)
*In vitro*	Pachymic acid	Bladder Cancer	EJ	Human	↑PARP, ↑ROS, ↑DR5, ↑Bax, ↓Bcl-2	0 μM–30 μM	24 h	20 μM	Negative	—	Jeong et al. (2015)
*In vitro*	Pachymic acid	Nasopharyngeal Carcinoma	CNE-1/CNE-2	Human	↑p-ATM, ↑p-ATR, ↑P-Chk-1, ↑P-Chk-2	0 μM–30 μM	72 h	CNE-1: 13.2 μM CNE-2: 4.8 μM	Negative	—	Zhang et al. (2017)
*In vitro*	Pachymic acid	Gallbladder Cancer	GBC-SD	Human	↓PCNA, ↓RhoA, ↓ICAM-1, ↓p-ERK1/2	10 µg/mL-50 μg/mL	48 h	10 μg/mL	Negative	—	Chen et al. (2015)
*In vivo*/*In vitro*	Pachymic acid	Lung Cancer	NCI-H23/NCI-H460	Human	↑ROS, ↑JNK, ↑ER.	*In vivo*:10, 30, 60 mg/kg, *In vitro*:0 μM–160 μM	*In vivo*-3weeks(5 day/week) *In vitro*-24 h	20 µM	Negative	—	Ma et al. (2015)
*In vitro*	Polyporenic acid C	Lung Cancer	A549	Human	↓PI3-kinase/Akt	0 μM–200 μM	72 h	6 μM	Negative	*Poria cocos* mushroom kernel	Ling et al. (2009)
*In vitro*	Poricoic acid A/B	Liver Cancer	HepG2	Human	↑ROS, ↓COX-2. ↓CDK1, ↓MMP-9	0 µg/mL-100 μg/mL	72 h	25 μg/mL	Positive	*Wolfiporia cocos* surface layer	Yue et al. (2023)
*In vivo*	Polysaccharide derivatives	Liver Cancer	HepG2/S-180	Human	↑Bax, ↓Bcl-2	20 mg/kg	8days	0.005 mg/mL	Negative	*Wolfiporia cocos* mycelia	Huang et al. (2006)
*In vitro*	Pachymic acid	Cervical Carcinoma	Caski	Human	↓CyclinD1, ↓TRIM9, ↓GSK-3β, ↓C-Myc	0 μmol/L-20.0 μmol/L	48 h	2.5 μmol/L	Negative	*Wolfiporia cocos* mushroom kernel	Shen and Weng (2020)
*In vitro*	Pachymic acid	Osteosarcoma	HOS	Human	↑PTEN, ↓p-Akt	0 μg/mL-50 μg/mL	72 h	10 μg/mL	Negative	—	Wen et al. (2018)
*In vitro*	Pachymic acid	Ovarian Cancer	HO-8910	Human	↑E-cadherin, ↓COX-2, ↓ β-catenin	0.5μM–2 μM	72 h	0.5 μM	Negative	—	Gao et al. (2015)
*In vivo*/*In vitro*	Poricoic acid A	Ovarian Cancer	SKOV3	Human	↑LC3-I, ↑LC3-II.	*In vivo*:10 mg/kg,*In vitro*: 0 μg/mL-80 μg/mL	*In vivo*-6weeks *In vitro*-24 h	30 μg/mL	Negative	—	Ma et al. (2021b)
*In vitro*	Pachymic acid	Prostate Cancer	LNCaP/DU145	Human	↓Bad, ↓Bcl-2	0 μg/mL-40 μg/mL	48 h	10 μg/mL	Negative	*Wolfiporia cocos* mushroom kernel	Gapter et al. (2005)
*In vitro*	Polysaccharide	Breast Cancer	MDA-MB-231	Human	↓SATB1	50 mg/L-200 mg/L	20 h	100 mg/L	Negative	—	Hu et al. (2019)
*In vitro*	Pachymic acid	Breast Cancer	MDA-MB-231/MCF-7	Human	↓PMA, ↓MMP-9	0 μM–30 μM	48 h	—	Negative	—	Ling et al. (2011)
*In vivo*/*In vitro*	Pachymic acid	Breast Cancer	MDA-MB-231	Human	↑PARP, ↓CyclinD1, ↓CDK2, ↓CDK4, ↓Bcl-2/Bax	*In vivo*:700 mg/kg, *In vitro*: 5 μg/mL-150 μg/mL	*In vivo*-25 days *In vitro*-96 h	5 μg/mL	Negative/Positive	the ethanol extract of *Wolfiporia cocos*	Jiang and Fan (2020)
*In vitro*	Pachymic acid	Squamous Carcinoma Of Tongue	CAL-27	Human	↑PARP, ↓CyclinD1, ↓CDK2, ↓CXCR4	2 μmol/L-8 μmol/L	48 h	2 μmol/L	Negative	—	Fan et al. (2021)
*In vivo*/*In* *vitro*	Pachymic acid	Kidney Cancer	A498	Human	↑TP53INP2, ↑TRAF6	*In vivo*: 30/60 mg/kg, *In* *vitro*: 0 μM–80 μM	*In vivo*-28 days *In vitro*-72 h	20 μM	Negative	—	Li et al. (2024)
*In vitro*	Pachymic acid	Gastric Cancer	—	Human	↓MMP2, ↓MMP-9, ↓TIMP1	0 μmol/L-160 μmol/L	28 h	20 μmol/L	Negative	—	Wang et al. (2022)
*In vivo*/*In vitro*	Pachymic acid	Gastric Cancer	MKN-49P/SGC-7901	Human	↑PPAR, ↓JAK2, ↓HIF1α, ↓Bcl-2/Bax, ↓STAT3	*In vivo*: 60 μM, *In vitro*: 60 mg/kg	*In vivo*-10 days *In vitro*-48 h	—	Negative	—	Lu et al. (2018)
*In vitro*	Pachymic acid polyporenic acid C Dehydropachymic acid	Pancreatic Cancer	PANC-1/MIA PaCa-2/AsPc-1/BxPc-3	Human	↓KRAS, ↓MMP-7	0 µg/mL-80 μg/mL	72 h	Panc-1: 24.5 μg/mL MiaPaca-2: 23.0 μg/mL AsPc-1: 11.3 μg/mL BxPc-3: 1.0 μg/mL	Negative	*Wolfiporia cocos* mushroom kernel	Cheng et al. (2013)
*In vivo*/*In vitro*	Pachymic acid	Pancreatic Cancer	PANC-1/MIA PaCa-2	Human	↑XBP-1s, ↑ATF4, ↑Hsp70, ↑CHOP, ↑p-eIF2α	*In vivo*: 25/50 mg/kg, *In vitro*: 0 μM–30 μM	*In vivo*-5weeks *In vitro*-24 h	15 μM	Negative	—	Cheng et al. (2015)
*In vitro*	Dehydroeburicoic acid	Ovarian Cancer	A2780	Human	↓MAPKs - caspase3	10–100 μM	24 h	—	Positive	*Wolfiporia cocos* mushroom kernel	Lee et al. (2017a)

### 3.2 Regulation of intestinal flora

The gut microbiota is the largest microbial community in the host’s body, known as the 'invisible organ of the human body'. The metabolic capacity of the human gut microbiota is an important factor in affecting nutrient absorption, immune regulation, the maintenance of health and the triggering of disease (Miao et al., 2016). Studies have demonstrated that carboxymethyl Poria polysaccharides (CMP) extracted from *Wolfiporia cocos* significantly mitigate colon damage induced by 5-fluorouracil (5-FU). This protective effect is associated with the inhibition of reactive oxygen species (ROS) production, an increase in the levels of catalase (CAT), glutathione peroxidase (GSH Px), and glutathione (GSH), as well as a reduction in the expression of pro-inflammatory markers such as NF-κB, p-p38, and Bax. Simultaneously, CMP enhances the expression of the antioxidant factors Nrf2 and Bcl-2. Moreover, CMP is effective in ameliorating gut microbiota dysbiosis caused by 5-FU, promoting an increase in the proportions of beneficial taxa such as Bacteroidetes, lactobacilli, butyrate-producing bacteria, and acetate-producing bacteria, while restoring overall gut microbiota diversity (Wang et al., 2018). Another investigation indicated that CMP can alleviate the cytotoxic effects of 5-FU, while concurrently enhancing the expression of tight junction proteins and related adhesion molecules, thus strengthening the intestinal barrier against GC (Yin et al., 2022). Yu et al. (2022) reported that *Poria cocos* polysaccharides (PCP) alleviate Chronic Non-Bacterial Prostatitis by modulating gut microbiota. Notably, after fermentation by the human gut microbiota, there was significant enrichment of Parabacterioides, Fusicatenibacter, and Parasutterella. These bacteria metabolize PCP to produce Haloperidol glucuronide and 7-ketodeoxycholic acid, which promote the expression of Alox15 and Pla2g2f in colon epithelium, while downregulating Cyp1a1 and Hsd17b7, thereby inhibiting inflammatory responses. This suggests that the metabolites Haloperidol glucuronide and 7-ketodeoxycholic acid may act as signaling molecules within the gut-prostate axis.

Lai et al. (2022) found that the water-soluble polysaccharide (PCX), water-insoluble polysaccharide (PCY) and triterpenoid saponin (PCZ) in *Poria cocos* can increase the number of lactobacilli in the intestine and change the content of short chain peptides in intestinal metabolites. Another study found that PCX, alkali soluble polysaccharide and triterpenoid acids have a protective effect on cisplatin induced intestinal injury, mainly by reducing the relative abundance of pathogenic bacteria such as *Proteus mirabilis*, cyanobacteria, ruminococcaceae and spirobacteriaceae, and promoting the growth of probiotics such as erysipelotticaae and prevotelacae (Zou et al., 2021). Lai et al. (2023) found that PCX can lower levels of inflammatory cytokines TNF-α and IL-1β, decrease the infiltration of inflammatory cells, and improve intestinal mucosal integrity and barrier function. This was achieved by increasing the relative abundance of beneficial gut microbiota and reducing harmful microbial populations, as manifested by elevated short-chain fatty acid (SCFAs) levels.

Xu et al. (2019) found through experiments that 16α - hydroxytrametinoic acid extracted from *Wolfiporia cocos* activates glucocorticoid receptor agonists, inhibits the activation of PI3K and Akt, to reduce the phosphorylation of downstream IκB and NF-κB, effectively alleviate TNF - α induced barrier damage in Caco-2 monolayer intestinal epithelial cells. This provides an improved strategy for adjuvant dietary therapy to restore intestinal health. Duan et al. (2023) upregulated the expression of intestinal Occludin and ZO-1, downregulated serum endotoxin, DAO, D-lactate, and intestinal myeloperoxidase (MPO) levels by extracting PCP, enhanced intestinal physical barrier, and increased the expression of MUC2, β-resistin, and SIgA in intestinal tissue, to enhance intestinal biochemical barrier. This indicates that PCP can be used as a functional food to regulate intestinal mucosal function, thereby improving the health of the intestine and host. Moreover, research has found that PCP can not only improve intestinal mucosal barrier function but also increase the diversity of intestinal microbiota to improve antibiotic associated diarrhea in mice (Xu et al., 2023). Table 5 summarizes the bioactivities of *Wolfiporia cocos* extraction in regulating of intestinal flora.

**TABLE 5:** Regulation of intestinal flora activities in *Wolfiporia cocos* extraction.

Model used	Extracts metabolites	Cell line/Model	Human/Mice cell	Activities	Dose range tested	Duration	Control	References
*In vivo*	Carboxymethylated pachyman	Colon cancer CT26	Mice	Increases the proportion of Bacteroidetes, lactobacilli, butyrate producing bacteria, acetate producing bacteria and SCFAs levels	25 mg/kg	14 days	Negative/Positive	Wang et al. (2018)
*In vivo*	Poria cocos polysaccharides	ApcMin/+ mice	—	Increases intercellular adhesion protein complexes and beneficial bacteria and reduces potentially pathogenic bacteria	40 mg/kg	4 weeks	Negative/Positive	Yin et al. (2022)
*In vivo*	Water-insoluble polysaccharide	C57BL/6	Mice	Increase in norank_f__Muribaculaceae, unclassified_f__Lachnospiraceae abundance and SCFAs. decrease in *Escherichia* - *Shigella*, *Staphylococcus* and *Acinetobacter*	300 mg/kg	10 days	Negative	Lai et al. (2023)
*In vivo/In vitro*	Poria cocos polysaccharides	Sprague-Dawley mice	—	Increase Parabacteroides, Fusicatenibacter and Parasutterella	*In vivo:* 250 mg/kg *In vitro*: Male fecal fermentation	*In vivo:* 28 days *In vitro*: 8 h	Negative	Yu et al. (2022)
*In vivo*	Water-soluble polysaccharides, Water-insoluble polysaccharides, Triterpenoid saponins	—	—	Increase lactic acid bacteria and SCFAs levels	PCX: 300 mg/kg, PCY: 300 mg/kg, PCZ: 150 mg/kg	15 days	Negative	Lai et al. (2022)
*In vivo*	Poria powder, Water - soluble polysaccharides, Alkali - soluble polysaccharides, Triterpene acids	C57BL/6	Mice	Decrease in Proteobacteria, Cyanobacteria, Ruminococcaceae and Helicobacteraceae. Increase in Erysipelotrichaceae and Prevotellaceae	PP: 2.0 g/kg, WP: 7.6 mg/kg, AP: 1.3 g/kg, TA: 6.0 mg/kg	13 days	Negative	Zou et al. (2021)
*In vitro*	16α - Hydroxytrametenolic acid	Caco – 2/293T/RAW 264.7	Mice	Inhibition of PI3K/Akt/NF-κB signaling pathway	10 μM–80 μM	24 h	Negative/Positive	Xu et al. (2019)

### 3.3 Antioxidation activity

Oxidation refers to the chemical reaction process between substances and oxygen, oxidative stress is a pathological state in which the redox homeostasis of an organism is imbalanced. It arises from the excessive production of reactive nitrogen species and ROS by the organism when subjected to external or internal stimuli, thereby breaking the original dynamic balance mechanism (Tabei et al., 2023). There are reports proving that supplementing exogenous antioxidants can eliminate free radicals and delay disease progression (Rahbari et al., 2015). However, artificially synthesized antioxidants are harmful to human health, such as liver damage and gout (Wang et al., 2016). Therefore, in this era of pursuing health and wellness, it is necessary to develop natural antioxidants to replace the current artificially synthesized antioxidants.

Recent experimental results have shown that the antioxidant capacity of hydroxymethyl PCP derivatives (PCP-C1, PCP-C2, PCP-C3) is directly related to the degree of carboxymethylation. The results showed that these derivatives possessed free radical scavenging and ferrous ion chelating efficacy, among which PCP-C3 protected renal cells from oxalate-induced oxidative damage, increased cell viability and antioxidant enzyme activities, and reduced the accumulation of harmful oxidative stress products. This suggests that PCP-C3 is a potential anticholinergic drug with great potential (Li CY. et al., 2021). Zhao et al. (2020) found that PCP effectively alleviated oxidative stress induced by oxidised low-density lipoprotein (oxLDL) by decreasing ROS and malondialdehyde (MDA) levels in vascular smooth muscle cells, while increasing superoxide dismutase (SOD) activity. By activating the ERK1/2 signalling pathway, the translocation of Nrf2 and the expression of heme oxygenase-1 were promoted, and the upregulation of Lectin-like oxidised LDL receptor-1 (LOX-1) was inhibited to reduce the uptake of oxLDL, which enhanced the antioxidant capacity of the cells. Fang et al. (2021) found that *Wolfiporia cocos* extract significantly reduced oxidative stress caused by ROS such as hydrogen peroxide, thereby inhibiting the activity of matrix metalloproteinases and reducing the degradation of collagen. At the same time, it can also upregulate the level of transforming growth factor beta 1 (TGF-β1), promote the regeneration and repair of skin cells, enhance the expression of antioxidant related proteins, and further enhance the antioxidant capacity of skin. This indicates that *Wolfiporia cocos* extract effectively delays the process of skin aging, providing the strong scientific basis for the development of new anti-aging cosmetics.

Wu et al. (2020) demonstrated through experiments that PCP has significant reducing and good scavenging abilities against DPPH, superoxide anions and hydroxyl radical and may be one of the main material bases for its antioxidant properties. Tang et al. (2014) found that PCP derivatives (PCP-1, PCP-2, and PCP-3) exhibit the ability to scavenge hydroxyl radicals and ABTS radicals, and they function through chelation of ferrous ions, thereby reducing the concentration of free ferrous ions and inhibiting oxidative stress responses. Xu et al. (2020) found that *Wolfiporia cocos*, an ingredient in Bajitianwan (BJTW), can reduce malondialdehyde (MDA) levels in the brain while simultaneously increasing the concentrations of catalase (CAT) and glutathione peroxidase (GSH Px) in serum. This dual action not only mitigates oxidative stress but also facilitates the upregulation of Forkhead box O1 (FoxO1) expression in bone tissue and enhances the levels of superoxide dismutase 2 (SOD2), thereby providing protection to both the bone and nervous system from oxidative damage. This suggests that BJTW has great potential in the treatment of Alzheimer’s disease and osteoporosis. Table 6 summarizes the bioactivities of *Wolfiporia cocos* extraction in antioxidation.

**TABLE 6:** Antioxidant activities in *Wolfiporia cocos* extraction.

Model used	Extracts metabolites	Cell line/Model	Human/Mice cell	Activities	Dose range tested	Duration	Control	References
*In vitro*	Carboxymethylated, Poria cocos polysaccharides	—	—	Scavenging free radicals and chelating ferrous ions	20 μg/mL-100 μg/mL	24 h	Negative/Positive	Li et al. (2021a)
*In vitro*	Poria cocos polysaccharides	VSMCs	Human	Inhibition of oxidized low-density lipoprotein-induced oxidative stress	50 μg/mL-200 μg/mL	24 h	Negative	Zhao et al. (2020)
*In vitro*	Poria cocos polysaccharides	Hs68	Human	Scavenging of DPPH, superoxide anion and hydroxyl radicals	100 μg/mL-400 μg/mL	24 h	Negative	Wu et al. (2020)
*In vitro*	Poria cocos polysaccharides	—	—	Scavenging hydroxyl radicals, ABTS radicals and chelating ferrous ions	1 mg/mL 10 mg/mL	4 h	Negative/Positive	Tang et al. (2014)

### 3.4 Anti-inflammatory activity

Inflammatory responses are known to be present in various disease processes. A study reported that CMP could regulate the balance of pro-inflammatory and anti-inflammatory cytokines in intestinal tissues by decreasing the expression of pro-inflammatory cytokines (TNF-α, IL-1β, IL-6) and increasing the levels of anti-inflammatory cytokines (IL-10, TGF-β), significantly preventing inflammatory bowel disease in mice (Liu et al., 2018). Song et al. (2018) found that PCP inhibits RANKL induced osteoclastogenesis by suppressing the activity of NFATc1 and the phosphorylation of ERK and STAT3. This suggests that PCP prevents and attenuates pathological fractures caused by bone resorption by interfering with the signalling pathway, decreasing osteoclast differentiation, and reducing bone resorption. Wu et al. (2022) established a fungal infection-induced peritonitis (FIP) mouse model and observed that polysaccharide compounds significantly alleviated inflammatory infiltration and cellular apoptosis in the thymus and spleen tissues. This effect is attributed to the reduction of inflammatory cytokines such as TNF-α, IL-6, and IL-1β, effectively ameliorating the inflammatory response. Additionally, PCP was found to decrease the levels of oxidative stress markers, including malondialdehyde (MDA) and myeloperoxidase (MPO), thereby mitigating oxidative damage. Wang et al. (2024) established a mouse model of bleomycin (BLM)-induced pulmonary fibrosis and found that PA inhibited BLM-induced increases in NLRP3, ASC, IL-1 β, P20, and TXNIP, decreased the levels of pro-inflammatory factors (IL -6 and TNF- α), and increased the level of the anti-inflammatory cytokine IL-10 in mouse lung tissue. It also reduced the levels of hydroxyproline and MDA in lung tissue and increased the activities of superoxide dismutase and glutathione peroxidase.

Li W. et al. (2021) explored the potential protective mechanism of PCP on compulsory spondylitis by establishing in the ApoE^−/−^ mice model induced by high-fat diet, and found that PCP can inhibit the increase of serum inflammatory mediators and blood lipids. Through experiments, it was found that PCP can significantly reduce the release of inflammatory mediators TNF - α, IL-6, and NO in serum, thereby protecting blood vessels from inflammatory invasion and reducing the elevation of low-density lipoprotein cholesterol, triglycerides, and total cholesterol in blood lipids. It also inhibits the activation of TLR4/NF-κB pathway in the aorta and blocks the expression of MMP-2 and ICAM-1. This indicates that PCP can intervene in ankylosing spondylitis by reducing inflammatory factors and blood lipid levels. Gui et al. (2021) conducted experiments by establishing a mouse model of fecal - induced peritonitis. They discovered that PA effectively ameliorated the pathological changes in the lung tissue of rats with pneumonia. This was achieved by inhibiting the activation of the NF-κB and MAPK signaling pathways, thereby reducing the release of inflammatory cytokines. Simultaneously, PA could also inhibit cell apoptosis, which further protected the damaged tissues and promoted the resolution of inflammation. These findings revealed the therapeutic potential of PA in inflammatory diseases and provided a scientific basis for the development of new anti-inflammatory drugs. Wu et al. (2023b) established a mouse model of osteoarthritis (OA) and found that PA promotes the expression of SIRT6, which inhibits the activation of the NF-κB signaling pathway. This modulation leads to a reduction in the production of inflammatory mediators such as inducible nitric oxide synthase (iNOS) and prostaglandin E2 (PGE2), as well as the suppression of IL-1β-induced inflammatory responses. Additionally, PA was found to reverse the abnormal upregulation of matrix metalloproteinase-3 and platelet-activating factor-5 in OA chondrocytes, while also downregulating the expression of type II collagen and aggrecan. These findings indicate that PA holds significant potential for the treatment of osteoarthritis. Table 7 summarizes the bioactivities of *Wolfiporia cocos* extraction in anti-inflammatory.

**TABLE 7:** Anti-inflammatory activities in *Wolfiporia cocos* extraction.

Model used	Extracts metabolites	Cell line/Model	Human/Mice cell	Activities	Dose range tested	Duration	Control	References
*In vivo*	Poria cocos polysaccharides	FIP	—	Reduction TNF-α, IL-6, IL-1β levels	200 mg/kg, 400 mg/kg	21 days	Negative	Wu et al. (2022)
*In vivo*	Pachymic Acid	BLM	—	Decreases IL-6 and IL-1β levels. Increases IL-10 levels	25, 50,100 mg/kg	28 days	Negative/Positive	Wang et al. (2024)
*In vivo*	Poria cocos polysaccharides	HFD	—	Reduction TNF-α, IL-6, and NO levels	100 mg/kg, 200 mg/kg, 400 mg/kg	11weeks	Negative	Li et al. (2021b)
*In vivo/In vitro*	Pachymic acid	Osteoarthritis in mice	—	Reduction NO, PGE2, TNF-α, IL-6, iNOS, COX-2 release	*In vivo:* 50 mg/kg, *in vitro*: 20 μM	*In vivo:* 8weeks, *In vitro*:48 h	Negative	Wu et al. (2023b)
*In vitro*	coriacoic acid A, coriacoic acid B, dehydroeburiconic acid, eburicoic acid, poricoic acid C	RAW 264.7	Mice	Inhibition of iNOS, COX-2 and NF-κB protein levels and reduction of LPS-induced phosphorylation of IKKα and IκBα	50 μM–100 μM	24 h	Negative/Positive	Lee et al. (2017b)

### 3.5 Immunomodulation activity

*Wolfiporia cocos* has immunomodulatory effects, and its extract can be used as a natural immune agent. There are reports indicating that PCP can increase NO by activating the Ca (2+)/PKC/p38/NF - κ B signalling pathway, TNF-α, IL-1β, IL-6 and intracellular calcium level, thereby enhancing the immune response of RAW 264.7 macrophages (Pu et al., 2019). Liu et al. (2021) found that *Wolfiporia cocos* derivatives CMP-1 and CMP-2 have a triple helix structure, which can improve the secretion of NO, TNF - α, and IL-6 by increasing the expression of iNOS, TNF–α and IL-6 mRNA, and enhance the immune function of RAW 264.7 macrophages.

Liu et al. (2020) established a model of anthrax protective antigen (APA) by extracting polysaccharide PCP-I from *Wolfiporia cocos* as an immune adjuvant. They found that PCP-I not only significantly enhanced anthrax specific anti APA antibodies, toxin neutralizing antibodies, anti-APA antibody affinity, as well as IgG1 and IgG2a levels, but also increased the frequency of APA specific memory B cells, increased the proliferation of PA specific spleen cells, significantly stimulated IL-4 secretion, enhanced the activation of dendritic cells *in vitro*, and improved the survival rate of mice immunized with anthrax lethal toxins. This indicates that polysaccharide PCP-I extracted from *Wolfiporia cocos* can activate immune signalling pathways, trigger immune synergy, and provide more effective immune responses. PCP-I is a very promising immune adjuvant. Chao et al. (2021) discovered that tumulosic acid, poronic acid C, and three-epi dehydrotumulosic acid—components of lanostane triterpenoids extracted from *Wolfiporia cocos*—can significantly stimulate the secretion of IFN-γ by mouse spleen cells. Concurrently, these lanostane triterpenoids activate natural killer cells, enhancing non-specific (innate) immunity and promoting the Th1 immune response, which leads to increased IFN-γ secretion. Additionally, they reduce the secretion of IL-4 and IL-5, cytokines associated with allergic reactions and the Th2 immune response. This research demonstrates that extracts from *Wolfiporia cocos* have the ability to modulate the Th1/Th2 immune response, potentially reducing the incidence of allergic diseases and positioning them as promising candidates for the development of anti-allergic therapies.

Liu et al. (2022) found that PCP significantly increased the activity of four enzymes related to immunity and energy metabolism (phenoloxidase, glucose-6-phosphate dehydrogenase, hexokinase, and fatty acid synthase), thereby significantly enhancing the cellular immunity of silkworms, including the ability of hemocyte phagocytosis, microaggregation and spreading. This indicates that PCP can regulate the immune system by enhancing cellular immunity, modulating immune responses, and regulating the expression levels of physiological metabolism related genes. Zhang W. et al. (2023) found that the polysaccharides PCWPW and PCWPS from *Wolfiporia cocos* contain some fucose and mannose residues, which could interact with mannose receptor on the surface of macrophages. By experimentally treating the polysaccharides PCWPW and PCWPS with the inhibitors, the secretion of TNFα was inhibited and NF-κB and MAP. Table 8 summarizes the bioactivities of *Wolfiporia cocos* extraction in immunomodulation.

**TABLE 8:** Immunomodulation activities in *Wolfiporia cocos* extraction.

Model used	Extracts metabolites	Cell line/Model	Human/Mice cell	Activities	Dose range tested	Duration	Control	References
*In vitro*	Poria cocos polysaccharides	RAW 264.7	Mice	Increase NO and activation of Ca(2+)/PKC/p38/NF-κ B	—	72 h	Negative	Pu et al. (2019)
*In vitro*	Carboxymethyl pachymaran	RAW 264.7	Mice	Upregulation of mRNA expression of iNOS, TNF-α and IL-6	12.5 μg/mL- 400 μg/mL	24 h	Negative/Positive	Liu et al. (2021)
*In vivo/In vitro*	Polysaccharide PCP-I	J774A.1/BMDCs	Mice	Activation of T cells and IL-4 secretion	—	68 h	Negative/Positive	Liu et al. (2020)
*In vivo*	lanostane Triterpenoids	BALB/c	—	Stimulation of IFN-γ and inhibition of the Th2 response	2.5, 5, 10, 20 mg/kg	9weeks	Negative/Positive	Chao et al. (2021)
*In vivo*	Poria cocos polysaccharides	*Bombyx mori*	—	Regulation immune signal recognition	0.1, 0.2, 0.4 μg/larval	24 h	Negative	Liu et al. (2022)
*In vitro*	PCWPW/PCWPS	RAW264.7	Mice	Activates MAPK, NF-κB and promotes TNF-αsecretion, mRNA expression	200, 400, 800 μg/mL	24 h	Negative/Positive	Zhang et al. (2023a)

### 3.6 Regulation of glycolipid metabolism

*Wolfiporia cocos* regulates metabolism mainly by regulating glucose and lipid metabolism disorders. Glucose metabolism is a complex process of sugar synthesis and decomposition in the body, and abnormal enzymes and other factors involved in synthesis and metabolism will lead to glucose metabolism disorders (Zhang et al., 2022). Genetic, environmental, or pathological conditions can lead to abnormal levels of blood lipids and lipoproteins, resulting in lipid metabolism (Badmus et al., 2022). Studies have shown that crude extracts of *Wolfiporia cocos* and its triterpenoids such as dehydrotumulosic acid, dehydrotrametinonic acid and pachymic acid can significantly reduce postprandial blood glucose in db/db mice. Further studies on a mouse model treated with streptozotocin showed that the crude extract of *Wolfiporia cocos* and triterpenoids exhibited insulin sensitizing activity, but not insulin releasing activity. This suggests that the active ingredients of *Wolfiporia cocos* may enhance insulin sensitivity through a pathway that is not dependent on PPAR-γ, thereby reducing blood glucose levels (Li et al., 2011).

Hyperlipidemia is an important factor leading to atherosclerosis. Some experimental studies have proved that after treatment with *Wolfiporia cocos*, hyperlipidemia and related lipid metabolite abnormalities were significantly improved (Miao et al., 2016). Kim et al. (2019) found that *Poria cocos* Wolf (PCW) extract can effectively improve liver steatosis. *In vitro* HepG2 cell experiments and *in vivo* high-fat diet mouse models, it was found that PCW can significantly reduce triglyceride levels in cells and mouse liver while affecting the expression of genes related to fat production, fatty acid oxidation, endoplasmic reticulum stress, and autophagy. PCW reduces fat production and promotes fatty acid oxidation by activating AMPK and its downstream pathways while inhibiting endoplasmic reticulum stress and inducing autophagy. These findings indicate that *Wolfiporia cocos* has the potential to be used for the treatment of hepatic steatosis. Sun et al. (2019) found that PCX extracted from the sclerotia of *Wolfiporia cocos* can significantly enhance glucose and lipid metabolism, as well as reduce liver steatosis in ob/ob mice. The mechanism of action for PCX involves increasing the abundance of butyrate-producing bacteria in the intestine, which in turn elevates intestinal butyrate levels, enhances the integrity of the intestinal mucosa, and activates the intestinal PPAR-γ pathway. Zhu et al. (2022) by establishing a high-fat diet (HFD) - induced obese mouse model, it was found that *Wolfiporia cocos* oligosaccharides(PCO) can reverse the imbalance of gut microbiota and changes in microbial metabolites, repair the intestinal barrier, reduce hyperglycemia, glucose tolerance, and insulin resistance in HFD mice, decrease the size of adipocytes, inhibit fat accumulation, and improve the disorder of glucose and lipid metabolism. This indicates that PCO, as a novel prebiotic, has great potential in the treatment of glucose and lipid metabolism diseases. Wang et al. (2023) found that CMP can significantly reduce fat weight and serum lipids, improve glucose tolerance, effectively reduce lipid droplet content in liver tissue, and promote cholesterol and lipid metabolism by reducing the synthesis of liver bile acids. They also found that CMP regulates the metabolism of glucose and lipid and energy balance by enhancing the abundances of Bifidobacterium, *Bacteroides*, and Akkermansia intestinal microbiota. Pan et al. (2023) found that *Wolfiporia cocos* acid can alleviate lipid metabolism disorders in mouse primary liver cells induced by OA-palmitic acid by activating SIRT6 signalling pathway. By using molecular docking, it was found that SIRT6/PPAR - α can promote fatty acid oxidation and SIRT6/Nrf2 can enhance antioxidant activity. The interaction between the two is a new target for the treatment of non-alcoholic fatty liver disease. Table 9 summarizes the bioactivities of *Wolfiporia cocos* extraction in regulating of glycolipid metabolism.

**TABLE 9:** Regulation of glycolipid metabolism activities in *Wolfiporia cocos* extraction.

Model used	Extracts metabolites	Cell line/Model	Activities	Dose range tested	Duration	Control	References
*In vivo/In vitro*	Dehydrotumulosic acid, Dehydrotrametenolic acid, Pachymic acid	db/db/C57BL mice	Enhancement insulin sensitivity to lower blood sugar	*In vivo*: 1, 5, 10 mg/kg. *In vitro*: 10、40、100 μM	24 h	Negative/Positive	Li et al. (2011)
*In vivo*	Wolfiporia powder	HLA mice	Regulation of fatty acid and sterol lipid metabolism	250 mg/kg	6weeks	Negative	Miao et al. (2016)
*In vivo/In vitro*	Poricoic acid, Pachymic acid Ergosterol	HepG2/C57BL/6 mice	Inhibition lipogenesis and stimulates fatty acid oxidation	*In vivo*: 100,300 mg/kg, *In vitro*: poricoic acid: 6.25–100 μM, pachymic acid/ergosterol: 0.63–10 μM	*In vivo*: 6weeks, *In vitro*: 24 h	Negative	Kim et al. (2019)
*In vivo*	Water insoluble polysaccharide	ob/ob mice	Improvement of intestinal mucosal integrity and activation of intestinal PPAR-γ pathway	1 g/kg^-1^, 0.5 g/kg^-1^	4 weeks	Negative/Positive	Sun et al. (2019)
*In vivo*	Poria cocos oligosaccharides	HFD mice	Regulation of BAs, SCFAs and tryptophan metabolites	200 mg/kg	16 weeks	Negative	Zhu et al. (2022)
*In vitro*	Pachymic acid	MPHs	Promotion fatty acid oxidation and reduces lipid deposition	12 μM–50 μM	24 h	Negative	Pan et al. (2023)

### 3.7 Improvement of organ function

Through research, it has been found that the active ingredients in *Wolfiporia cocos* have the ability to improve the function of human organs such as the heart (Xie et al., 2023), liver (Jiang et al., 2022) and kidneys (Wu et al., 2023a). Table 10 summarizes the bioactivities of *Wolfiporia cocos* extraction in improving of organ function.

**TABLE 10:** The mechanism of improving organ function in *Wolfiporia cocos* extraction.

Model used	Extracts metabolites	Cell line/Model	Human/Mice cell	Mechanism	Dose range tested	Duration	Control	References
*In vivo*	Pachymic acid	HS mice	—	Inhibition of cardiomyocyte apoptosis	7.5, 15 mg/kg	3days	Negative	Liu et al. (2023)
*In vivo*	Poria cocos polysaccharides	MI/RI mice	—	Inhibition of ROS production thereby reducing cardiomyocyte apoptosis	100, 200 mg/kg	7days	Negative/Positive	Xie et al. (2023)
*In vitro*	Pachymic acid	H9c2	Human	Reduces TNF-α, IL-1, and IL-6 release and inhibits apoptosis in cardiomyocytes	0.125–20 μM	24 h	Negative	Li et al. (2015)
*In vivo*	Poria cocos polysaccharides	NASH	—	Inhibition NF - κB activation and CCL3/CCR1 mRNA expression. Protects liver tissue	150, 300 mg/kg	4 weeks	Negative	Tan et al. (2022)
*In vivo*	Poria cocos polysaccharides	Gao-Binge	—	Inhibition the CYP2E1/ROS/MAPKs signaling pathway. Ameliorates apoptosis in liver cells	25, 50, 100 mg/kg	16 days	Negative/Positive	Jiang et al. (2022)
*In vivo/In vitro*	Poria cocos polysaccharides	APAP/AML12	Mice	Decrease TNF-β and TNFsR-Ⅰ levels. Reduces hepatocyte inflammation	*In vivo*: 200, 400 mg/kg, *In vitro*: 20, 40 g/L	*In vivo*:14days, *In vitro*:48 h	Negative/Positive	Wu et al. (2019)
*In vivo*	Poria cocos polysaccharides	APAP	—	Decrease serum levels of TNF-α, IL-6, and increase expression of AKR7A, c-Jun, and Bcl-2 in liver tissue	200, 400 mg/kg	14days	Negative	Wu et al. (2018)
*In vivo/In vitro*	Poricoic acid A	UUO/NRK-49F	Mice	Inhibition twist, snail1, MMP-7, and PAI-1. reduces renal fibroblast production	*In vivo*: 10 mg/kg, *In vitro*: 10 μM	*In vivo*: 2weeks, *In vitro*: 48 h	Negative/Positive	Chen et al. (2023)
*In vivo/In vitro*	Poricoic acid A	DKD/MPC5	—	Increase LC3 and ATG5 levels and decrease p62 and FUNDC1 levels. Reduces kidney injury	*In vivo*: 10, 20 mg/kg, *In vitro*:0 μg/mL-200μg/mL	*In vivo*: 4 weeks, *In vitro*: 24 h	Negative	Wu et al. (2023a)
*In vitro*	Poricoic acid A	TGF-β1/NRK-49F	Mice	Inhibit PDGF-C, Smad3 and MAPK signaling pathways. Reduce renal fibroblast proliferation	1μM–20 μM	24 h	Negative	Li et al. (2021c)
*In vivo*	Pachymic acid	CKD	—	Upregulates renal klotho levels and inhibits the Wnt/β - catenin signaling pathway. Reduces renal inflammation	10 mg/kg	4 weeks	Negative/Positive	Younis et al. (2022)
*In vitro*	Poricoic acid ZA	TGF-β1/ANGII	—	Inhibition the renin-angiotensin system and the TGF-β/Smad signaling pathway. Reduce renal fibrosis	10 μM		Negative/Positive	Wang et al. (2017)

#### 3.7.1 Improve heart function

A study has reported that by establishing a myocardial ischemia (MI/RI) rat model, *Wolfiporia cocos* polysaccharides reduce the levels of LDH, CK-MB, IL-1 β, IL-18, and MDA in myocardial tissue. At the same time, they reduce the relative expression levels of Bax, cleaved caspase-3, RhoA, ROCK1, and p-MYPT-1 proteins, as well as increase the relative expression levels of SOD and Bcl-2 proteins in myocardial tissue, thereby improving tissue edema and microcirculation disorders, and weakening pathological damage and myocardial cell apoptosis. Meanwhile, by downregulating the levels of RhoA, ROCK1, and downstream signalling factor p-MYPT-1 in MI/RI rat myocardial tissue, the activation of the Rho ROCK signalling pathway is inhibited, the activation of inflammasomes is reduced, and myocardial cell oxidation and inflammatory damage are alleviated, thereby reducing myocardial cell apoptosis (Xie et al., 2023). Liu et al. (2023) found that the triterpenoid compound PA extracted from *Wolfiporia cocos* can reduce the levels of IL-1 β, IL-6, and TNF-α by inhibiting the pro-inflammatory NF-κB signalling pathway, thereby improving hematopoietic shock (HS) - induced cardiac inflammation. Coincidentally, PA weakens the increase in HS induced cardiac monocyte/macrophage and neutrophil infiltration, as well as inhibits HS induced M1 polarization and exaggerates M2 polarization in myocardial tissue, reducing cardiac damage, inhibiting cell apoptosis, and improving cardiac inflammatory response. Li et al. (2015) found that PA exhibited significant effects in inhibiting lipopolysaccharide (LPS) - induced apoptosis and inflammatory response in H9c2 cardiomyocytes. Through PA treatment, the upregulation and release of TNF-α, IL -1, and IL-6 inflammatory factors in myocardial cells can be significantly reduced. At the same time, PA inhibits LPS induced myocardial cell apoptosis by suppressing the phosphorylation of extracellular regulated kinase (Erk) 1/2 and p38 signalling pathways. This discovery suggests that PA may be a potentially effective drug for treating LPS induced myocarditis and apoptosis, providing a new strategy for treating inflammation related cardiovascular diseases.

#### 3.7.2 Improve liver function

In the early stages, research on carboxy methyl *Poria cocos* polysaccharide (CMPCP) for chronic viral hepatitis has been conducted. Through experiments, it was found that CMPCP can improve liver function and enhance non-specific cell-mediated immune function, without cytotoxic effects. This study was a preliminary investigation of the use of *Wolfiporia cocos* in the treatment of liver diseases (Guo et al., 1984). With the constant evolution of social times, pressures and other factors have led to an increasing intake of alcohol, gradually making alcoholic liver disease (ALD) the leading chronic liver disease worldwide, placing a heavy burden on the global public health system (Zhang N. et al., 2023). There are research reports that the active *Poria cocos* polysaccharide (PCP-1C) improves ALD by inhibiting the TLR4/NF-κB and CYP2E1/ROS/MAPK pathways, repairing the intestinal barrier and reducing LPS leakage, thereby reducing liver injury, inflammation, oxidative stress, and intestinal leakage (Jiang et al., 2022). Tan et al. (2022) established a non-alcoholic steatohepatitis (NASH) model by administering methionine and choline deficiency diet to C57BL/6 mice for 4 weeks. They found that *Wolfiporia cocos* polysaccharides can reshape the composition of intestinal bacteria by significantly increasing the relative abundance of Faecalibaculum and reducing the endotoxin load level from intestinal bacteria. This suggests that *Wolfiporia cocos* polysaccharides can provide a new potential strategy for the prevention and treatment of NASH. Wu et al. (2019) demonstrated through experiments that PCP can reduce Hsp90 cells, be beneficial for acetaminophen-induced liver cell damage, and enhance its hepatoprotective effect. PCP (Wu et al., 2018) can alleviate liver injury in a dose-dependent manner by downregulating the expression of NF-κB/p65 and IkB α.

#### 3.7.3 Improve kidney function

Chen et al. (2023) found that inducing renal interstitial fibrosis in rats or mice by establishing unilateral ureteral obstruction (UUO), and PAA from *Wolfiporia cocos* can promote β-catenin K49 deacetylation, significantly inhibit renal fiber cell activation, and improve renal function. At the same time, Wu et al. (2023a) by establishing a model of diabetes nephropathy (DKD) and extracting PAA from *Wolfiporia cocos*, found that PAA can significantly reduce the levels of blood sugar and urinary protein in mice, control renal fibrosis, and downregulate FUNDC1 to promote mitosis, thus having a beneficial impact on the damage of capsular cells in DKD and effectively alleviating renal damage. There is experimental evidence (Li Q. et al., 2021) that PAA inhibits the PDGF-C, Smad3, and MAPK pathways to suppress TGF-β1 induced ECM accumulation, fibrosis formation, and proliferation in renal fibroblasts. Fu et al. (2022) found that *Wolfiporia cocos* polysaccharides can not only induce proliferation and differentiation of bone marrow mesenchymal stem cells, but also reduce the level of pro-inflammatory cytokines to improve kidney morphology, thereby improving chronic kidney disease. Younis et al. (2022) found through experiments that PA has an upregulation effect on renal klotho, thereby inhibiting Wnt/β - catenin reactivation and downregulating RAS gene expression, which brings benefits to the treatment of chronic kidney disease (CKD). At the same time, Wang et al. (2017) confirmed that Poricoic acid ZA extracted from *Wolfiporia cocos* is used as a renin-angiotensin system inhibitor for the treatment of CKD. It blocks the interaction between Smad2/3-TGF β RI proteins and inhibits Smad2/3 phosphorylation, thereby inhibiting RAS the TGF - β/Smad pathway, ultimately leading to the treatment of chronic kidney disease.

## 4 Toxicology

The “*Shennong Bencao Jing*” describes the traditional Chinese medicine derived from *Wolfiporia cocos* as being “sweet, smooth, and devoid of toxicity.” Modern studies have confirmed that the hydroalcoholic extract of *Wolfiporia cocos* has oral and topical anti-inflammatory activity in mice. Two metabolites isolated from it showed strong inhibitory effects and low toxicity on acute TPA edema, and the safe dosage is 6–18 g (Cuellar et al., 1997). The toxicological properties of the water-soluble heteropolysaccharide ac - PCM0 from *Wolfiporia cocos* were investigated by *in vivo* acute toxicity test and comparative experiments. The heteropolysaccharide solution with a concentration of 50 mg/mL was intravenously injected into BALB/C mice weighing 201 g. The toxicity and mortality were recorded for seven consecutive days. The LD50 of the polysaccharide was calculated to be higher than 1,250 mg/kg, indicating that the polysaccharide is non-toxic (Zhang et al., 2005). An *in vivo* toxicity assay was conducted to evaluate the potential toxicity of PAA during the treatment of T-ALL. T-ALL nude mice were randomly divided into three groups: control group, PAA low dose group (5 mg/kg) and PAA high dose group (10 mg/kg); NOD/SCID mice were divided into corresponding control group and PAA treatment group. The PAA treatment group was given an intraperitoneal injection, and the control group was given the same amount of solvent (physiological saline). After 4 weeks of treatment, it was detected that PAA had no significant effect on the levels of alt, AST, bun and Cr in serum. This indicates that PAA has no significant hepatotoxicity or nephrotoxicity (Chen et al., 2022).

## 5 Conclusion

In recent years, *Wolfiporia cocos* has attracted more and more attention from researchers, and many studies have also confirmed its medicinal value. In terms of active ingredients, polysaccharides and terpenoids have been the main research objects. Although researchers have made great efforts in elucidating their chemical structures and biological activities, there are still some limitations. As far as polysaccharides are concerned, the purification process is still a formidable challenge. Most natural polysaccharides are insoluble in water. Researchers mostly use crude extracts or derivatives, which makes the fine structure of polysaccharides unclear, and hinders the accurate understanding of its mechanism of action to a certain extent. It is hoped that the fine structure of *Wolfiporia cocos* polysaccharide can be described through more advanced technology improvement in the future. On the other hand, the terpenoids in *Wolfiporia cocos* are mainly triterpenoids, and also contain trace diterpenes. Most of the current research focuses on triterpenoids, while the research on diterpenes is relatively scarce. In the future, if the research on diterpenes can be strengthened, it may open up a new research path for revealing the pharmacological activity of *Wolfiporia cocos*, and provide a richer scientific basis for the in-depth development and wide application of *Wolfiporia cocos*.

**FIGURE 6:**
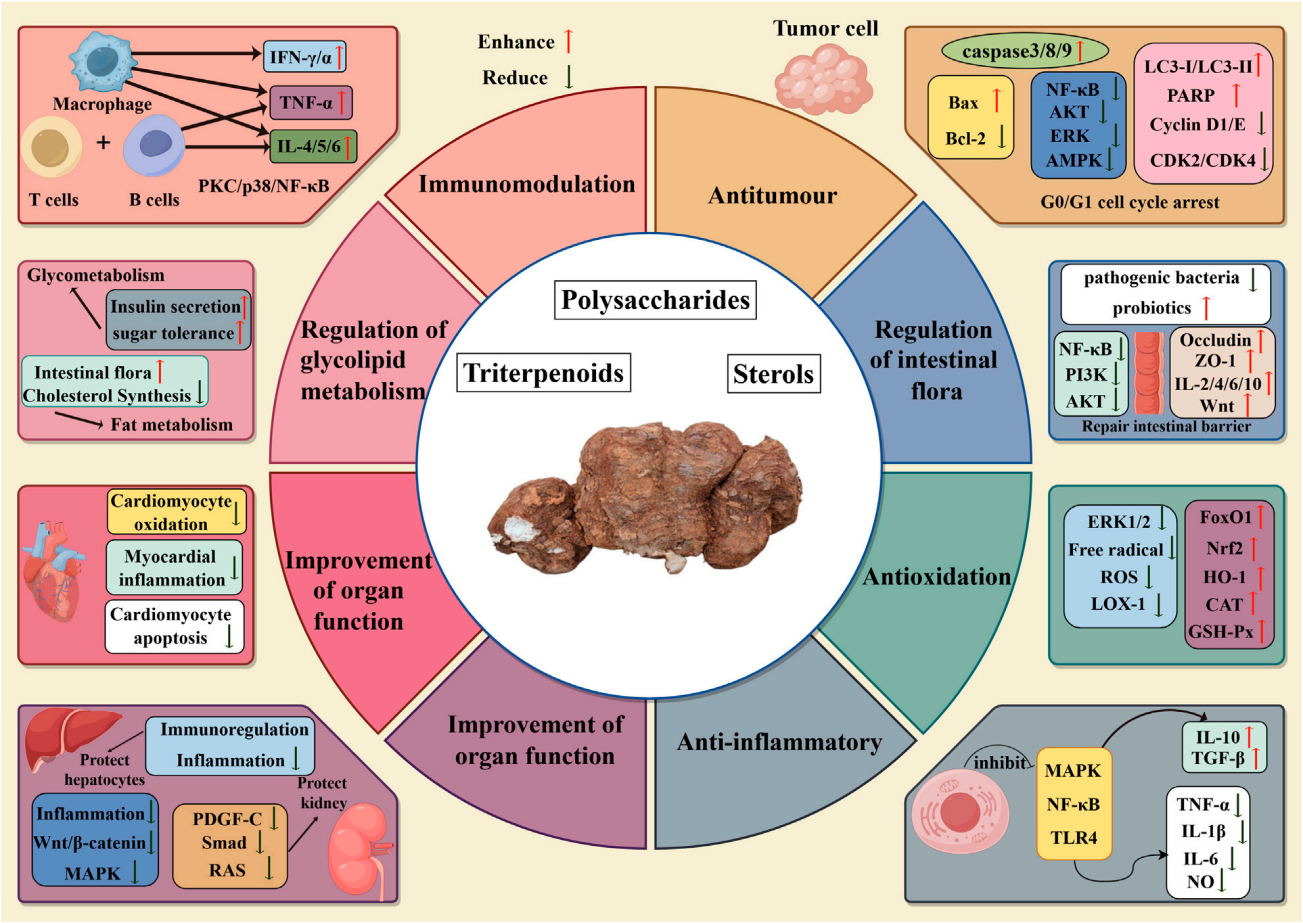
Mechanism diagram of biological activity of *Wolfiporia cocos*.

*Wolfiporia cocos*, as a traditional Chinese medicine with a wide range of pharmacological mechanisms, has demonstrated *in vitro* and *in vivo* experiments the potential for a wide range of applications such as antitumour, antioxidation, anti-inflammatory, immunomodulation, regulation of intestinal flora, regulation of glycolipid metabolism, and improvement of organ function. As shown in Figure 6. *In vitro* experiments showed that *Wolfiporia cocos* extracts have antitumour, antioxidation, anti-inflammatory and immunomodulation activities. In the *in vivo* model, the extract showed antitumour, regulation of intestinal flora, regulation of glycolipid metabolism, and improvement of organ function. Although *in vitro* experimental studies can precisely regulate the experimental conditions and thus obtain preliminary conclusions faster on the basis of controlled variables, it is difficult to comprehensively simulate the complexity of the *in vivo* environment, and it is more general for elucidating the mechanism of action of *Wolfiporia cocos* extracts in detail. As for *in vivo* experiments, more experiments are currently using mouse models to simulate human beings, although there are many similarities between mice and human beings in physiological mechanisms, mice are still unable to fully reflect the complexity of the human body *in vivo*. In the future, it is necessary to strengthen clinical research to promote *Wolfiporia cocos* from the laboratory to clinical application, so that it can truly benefit human health.

In conclusion, in order to provide inspiration for the further study of *Wolfiporia cocos*, this paper summarizes the research status of *Wolfiporia cocos* in chemistry, active ingredients and pharmacological mechanism. Although *Wolfiporia cocos* has shown significant application potential in many fields, its complex biological activity mechanism and fine chemical structure characteristics still need to be further explored and established, so as to fully explore its value in the development of functional food additives and drugs. On this basis, we suggest that the use of modern biotechnology, chemical analysis and computer science and other technologies, in-depth excavation of polysaccharide and terpenoids derivatives and other potential active ingredients in *Wolfiporia cocos*. Through this way, it is not only expected to find more new compounds with unique biological activities, but also further expand the application scope of *Wolfiporia cocos* in medicine, food, health products and other fields, laying a solid foundation for the maximum utilization of *Wolfiporia cocos* resources. We look forward to more researchers joining the research of *Wolfiporia cocos* in the future to jointly promote the modernization process of this traditional Chinese medicine.
